# Bioactivities and mechanisms of natural medicines in the management of pulmonary arterial hypertension

**DOI:** 10.1186/s13020-022-00568-w

**Published:** 2022-01-15

**Authors:** Zhijie Yu, Jun Xiao, Xiao Chen, Yi Ruan, Yang Chen, Xiaoyuan Zheng, Qiang Wang

**Affiliations:** 1 Pharmacy Department, Chongqing Emergency Medical Center, Chongqing University Central Hospital, Chongqing, 400014 China; 2 Department of Cardiovascular Medicine, Chongqing Emergency Medical Center, Chongqing University Central Hospital, Chongqing, 400014 China; 3 Department of Pharmacy, The Second Affiliated Hospital, Army Medical University, Chongqing, 400037 China

**Keywords:** Pulmonary arterial hypertension, Pathology, Pathogenesis, Natural medicines, Mechanisms, Clinical studies

## Abstract

Pulmonary arterial hypertension (PAH) is a progressive and rare disease without obvious clinical symptoms that shares characteristics with pulmonary vascular remodeling. Right heart failure in the terminal phase of PAH seriously threatens the lives of patients. This review attempts to comprehensively outline the current state of knowledge on PAH its pathology, pathogenesis, natural medicines therapy, mechanisms and clinical studies to provide potential treatment strategies. Although PAH and pulmonary hypertension have similar pathological features, PAH exhibits significantly elevated pulmonary vascular resistance caused by vascular stenosis and occlusion. Currently, the pathogenesis of PAH is thought to involve multiple factors, primarily including genetic/epigenetic factors, vascular cellular dysregulation, metabolic dysfunction, even inflammation and immunization. Yet many issues regarding PAH need to be clarified, such as the “oestrogen paradox”. About 25 kinds monomers derived from natural medicine have been verified to protect against to PAH via modulating BMPR2/Smad, HIF-1α, PI3K/Akt/mTOR and eNOS/NO/cGMP signalling pathways. Yet limited and single PAH animal models may not corroborate the efficacy of natural medicines, and those natural compounds how to regulate crucial genes, proteins and even microRNA and lncRNA still need to put great attention. Additionally, pharmacokinetic studies and safety evaluation of natural medicines for the treatment of PAH should be undertaken in future studies. Meanwhile, methods for validating the efficacy of natural drugs in multiple PAH animal models and precise clinical design are also urgently needed to promote advances in PAH.

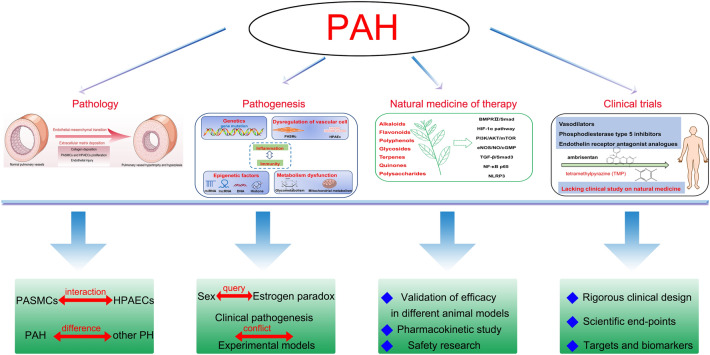

## Introduction

Pulmonary arterial hypertension (PAH), a subtype of pulmonary hypertension (PH), shares representative characteristics with pulmonary vascular remodelling and resistance, potentially resulting in right heart failure and death. The incidence of PH increases to 10% in aged 65 years and older in the world [1]. Meanwhile, epidemiological investigation demonstrated that PAH morbidity was approximately 15 cases per million due to its rarity and favoured young women, but with remarkable mortality [2]. Clinically, pulmonary artery wedge pressure (PAWP) ≤ 15 mmHg and pulmonary vascular resistance (PVR) > 3 Wood units comprise a diagnosis of PAH. Elevated pulmonary artery pressure is primarily ascribed to an imbalance in vasodilator factors and vasoconstrictor factors (nitric oxide (NO)/endothelin-1 (ET-1)) [3]. Currently, multiple pharmacotherapies have been developed based on the accumulated understand of the pathogenesis and targets of PAH, primarily including calcium channel blockers, prostaglandin analogues, endothelin receptor antagonists, phosphodiesterase inhibitors and soluble guanylate cyclase stimulators [4, 5]. Meanwhile, existing clinical trials also primarily focus on the abovementioned drugs. Interestingly, the abundance of natural medicines has also exhibited potent efficacy for PAH in both in vivo and in vitro experiments, but their clinical study for PAH treatment remains deficient [6]. Therefore, we systematically outline the current knowledge concerning the pathology, pathogenesis, therapeutic effects of natural medicine, their mechanism of action and clinical studies for PAH in this review, which provides a comprehensive summary and critical discussion for further understanding of PAH pathophysiology.

## Categories of PH

A classical clinical diagnosis of PH consists of a mean pulmonary arterial pressure (mPAP) ≥ 25 mmHg, PAWP ≤ 15 mmHg and PVR > 3 Wood units. Patients with PAH exhibit various clinical symptoms, such as fatigue, shortness of breath, tiredness, dizziness and chest pain [7]. Nevertheless, PH caused by different factors also exhibits distinct clinical features. For instance, the definition of hypoxic pulmonary artery hypertension (HPAH) is clinically diagnosed by the presence of a mean right ventricular pressure ≥ 30 mmHg [8]. According to the updated consensus of the Taiwan Society of Cardiology (TSOC) working group on pulmonary hypertension, PH primarily includes five categories: (a) pulmonary arterial hypertension; (b) pulmonary hypertension induced by left heart disease; (c) pulmonary hypertension due to lung disease or hypoxia; (d) chronic thromboembolic pulmonary hypertension (CTEPH); and (e) pulmonary hypertension with unclear aetiology [9, 10]. As shown in Table 1, multiple factors can cause PH, such as heredity, drugs, left ventricular dysfunction, chronic hypoxia, lung disease, pulmonary artery obstructions and others. Among them, PH induced by left heart disease is most prevalent, exhibiting up to 25% to 83% morbidity, implying that left ventricular systolic/diastolic dysfunction may play a critical role in the pathogenesis of PH [11]. Furthermore, patients with PH due to left heart disease are preferentially older and female [12]. Meanwhile, chronic hypoxia resulting in PH is also frequently observed in patients. Those with heritable PH largely present with gene mutations of bone morphogenetic protein receptor 2 (BMPR2), as well as newly identified caveolin-1, KCNK3, EIF2AK4 and TBXA2 [13–15]. However, reduced expression of caveolin-1 and KCNK3 might not participate in TGF-β signalling. Additionally, certain drugs and compounds, such as aminorex, fenfluramine, dexfenfluramine, benfluorex and toxic rapeseed oil, have been demonstrated to induce PAH [10]. It is commonly recognized that the vasoconstrictor factors (i.e. NO and ET-1) are the causes of primary PAH. Certainly, the specific subtypes of PAH are induced by different pathogenesis. Taken together, progressive PAH severely endangers patient lives due to its outcome of right heart failure, which occurs in the absence of representative clinical symptoms and precise diagnosis. Invasive right-heart catheterization has been regarded as mandatory clinical diagnostic criterion for PAH, but application of echocardiography might convey new insights into evaluating the diagnosis, treatment and prognosis of PAH [16]. The importance of echocardiography for evaluating right ventricular systolic function has been deemed essential to the prognosis of PAH. For instance, advanced three-dimensional echocardiography exhibits better reproducibility and agreement with cardiac magnetic resonance in right atrial volumes, right ventricular volumes and ejection fraction [17]. Additionally, computed tomography assessment for lung parenchyma, pulmonary arteries and heart may provide valuable information to confirm the specific subtype of PH [18]. Taken together, the precise clinical diagnosis and prognosis of PH still need to be further refined by advanced clinical methods. For example, the current deficiencies of electrocardiogram with less sensibility for PH, chest radiograph with weak evidence to diagnose PH and unstandardized parameters in echocardiography still need to be settled [9].

**Table 1:** Clinical categories of PH

Categories	Pathogenesis
Pulmonary arterial hypertension	Idiopathic; Heritable (BMPR2 or other mutation); Drug or toxin-induced PH; Connective tissue disease and HIV infection et al.; Schistosomiasis
Pulmonary hypertension due to left heart disease	Left ventricular systolic/diastolic dysfunction; Valvular disease et al
Pulmonary hypertension due to lung diseases or hypoxia	Chronic exposure to high altitude; Other lung diseases
Chronic thromboembolic pulmonary hypertension	Other pulmonary artery obstructions (angiosarcoma; arteritis; parasites; congenital pulmonary artery stenosis)
Pulmonary hypertension with unclear mechanism	Chronic haemolytic anaemia; Myeloproliferative disorders; Metabolic disorders; Others

## Pathophysiology of PAH

**Fig. 1:**
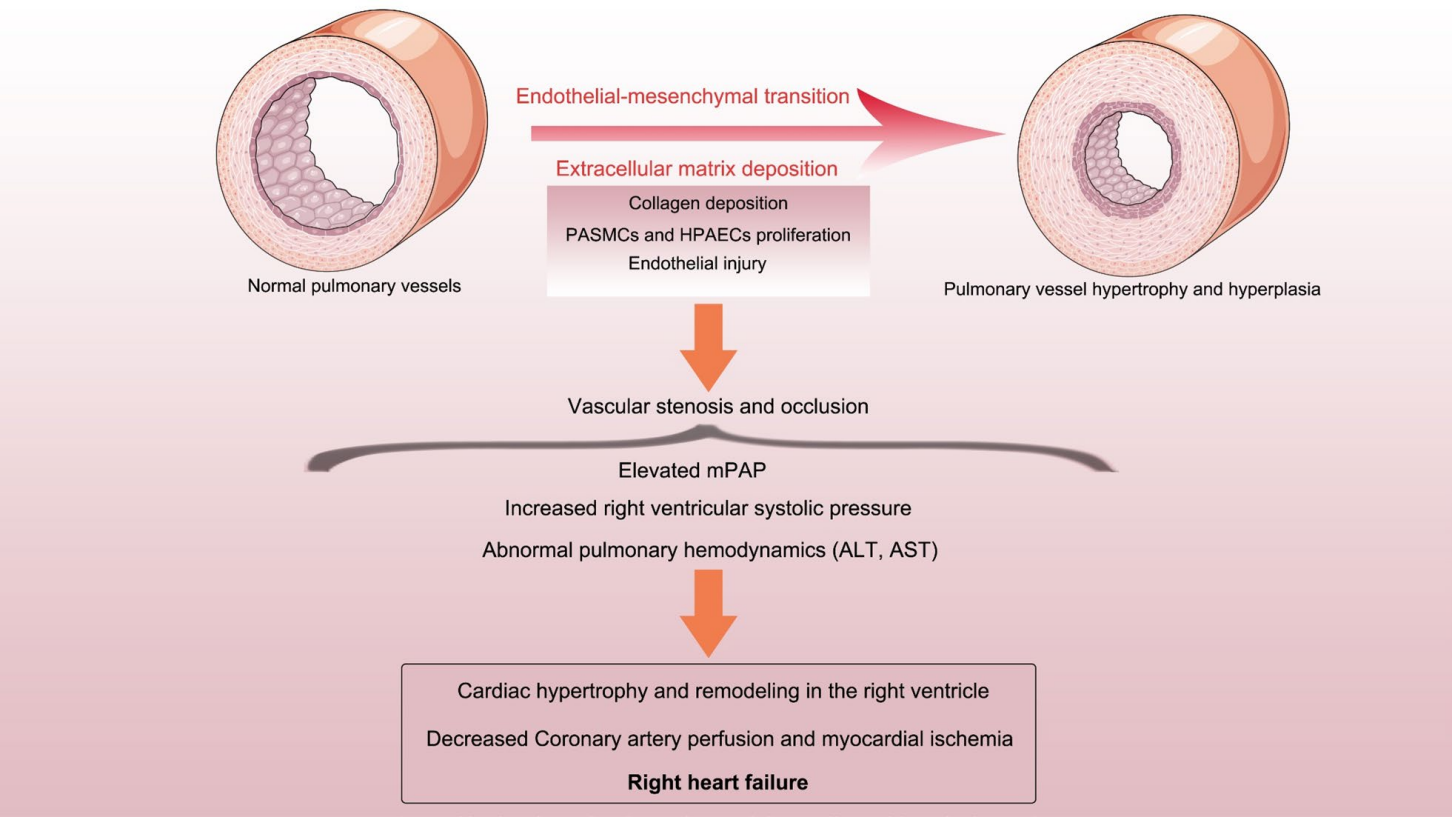
Schematic diagram of pulmonary vascular remodelling. Endothelial–mesenchymal transition, increased extracellular matrix and proliferation of vascular cells (PASMCs and HPAECs) induced vascular stenosis and occlusion, even further leads to right ventricular failure. *mPAP* mean pulmonary arterial pressure, *ALT* alanine aminotransferase, *AST* aspartate aminotransferase

PAH shows evident features of vascular remodelling in the pulmonary artery, primarily including increased extracellular matrix with collagen deposition, proliferation of pulmonary arterial smooth muscle cells (PASMCs) and human pulmonary arterial endothelial cells (HPAECs) in pulmonary vessels, and endothelial injury. These changes result in hypertrophy, stenosis, and even occlusion of the medial pulmonary vessels [19]. Therefore, PAH patients present with clinically elevated pulmonary arterial pressure and aberrant pulmonary haemodynamics. With the progressive development of PAH, myocardial cell remodelling and dysfunction in the right ventricle also appear [20]. Finally, aggravation of myocardial ischaemia further leads to right ventricular failure, which is largely responsible for premature death in PAH patients (Fig. 1). Meanwhile, the recovery of right heart function also affects the prognosis and survival rate in PAH patients after drug treatment. A previous revealed that the development of right ventricular (RV) failure was also associated with myocardial apoptosis, fibrosis, decreased RV capillary density, and a decreased vascular endothelial growth factor expression. Another finding indicated that RV failure associated with pulmonary hypertension is strictly due to increased RV afterload [21]. Interestingly, another study suggested that the type of pulmonary vascular remodelling caused by certain factors might be reversible. Due to the shift of PASMCs between proliferative and non-proliferative phenotypes, suggesting cellular plasticity, pulmonary vascular remodelling with medial thickening and muscularization might be reversible. In contrast, vascular remodelling induced by impaired apoptotic regulation of endothelial cells and endothelial damage is likely irreversible, especially when accompanied by congenital heart disease [22]. Determining how to improve cellular plasticity and muscularization in PASMCs to reverse pulmonary vascular remodelling may represent a new potential perspective for the treatment of PAH in future studies. Additionally, multiple studies have also demonstrated that the endothelial–mesenchymal transition (EndMT) caused by hypoxia was also a vital factor for pulmonary vascular remodelling in rats [23, 24]. At present, due to cardiopulmonary pathophysiology also contributing to neuroinflammation, altered gastrointestinal function, and increased bone marrow-derived cells, PAH could be considered a brain–gut–lung systemic disease [25].

## Pathogenesis of PAH

### Genetic and epigenetic factors

It is well recognized that mutations in BMPR2 in heritable PAH patients are very common. As one member of the transforming growth factor-β (TGF-β) superfamily, deletion of BMPR2 directly contributes to dysregulation of BMP signalling and cellular aberrations (e.g., abnormal proliferation, migration and extracellular matrix deposition) [26]. Currently, additional gene mutations have been continually identified in PAH patients by high-throughput sequencing, including activin A receptor type II-like 1 (ACVRL1), endoglin (ENG), and members of the Smad family (SMAD1, SMAD4 and SMAD9) [27]. Meanwhile, genetic variants in caveolin-1, potassium channel subfamily K member 3 (KCNK3) and eukaryotic translation initiation factor 2 alpha kinase 4 (EIF2AK4) have been implicated in the development of PAH through whole exome sequencing methods [28]. In fact, approximately 16 genetic risk factors leading to PAH have been identified. But mutations in these genes do not all convey the same risk for PAH. Undoubtedly, the most common BMPR2 mutation remains the predominant genetic predisposition responsible for PAH. Additionally, mutations in ACVRL1 and EIF2AK4 also exhibit high mutation frequencies in PAH positive patients, especially in female patients [29]. Nonetheless, increasing numbers of genetic abnormalities have been found to associate with PAH, but how these mutations affect transcription and translation to modulate protein expression and signalling remains poorly understood. Thus, additional studies are needed to clarify the molecular features and protein function of these genetic variants via genomics-related strategies both in vitro and in vivo. In addition, variation of those identified mutated gene whether and how to impact the diagnosis, drug management and prognosis of PAH still warrant further investigation.

Existing studies have determined that gene expression in PAH is also regulated by epigenetic factors, including DNA methylation, interference of microRNAs and histone modification, but these processes do not change the sequence of genes [30, 31]. In 2010, Archer et al.’s [32] investigation first demonstrated that epigenetic deficiency of superoxide dismutase (SOD)-2 due to gene methylation in an enhancer region of intron 2 and in the promoter triggered abnormal proliferation and apoptosis resistance in a heritable PAH rat model. The primary epigenetic mechanism of DNA methylation includes influencing the redox reaction via downregulating expression of SOD2 to reduce hydrogen peroxide levels; hydrogen peroxide then contributes to high expression of HIF-1α, even in rats with normoxia. At this point, the activated HIF-1α initiates the Warburg effect and regulates corresponding signalling pathways, resulting in PAH [33]. Second, increasing evidence suggests that regulation of microRNAs plays a crucial role in the development of PAH. To the best of our knowledge, microRNAs consist of a class of small non-coding RNAs that modulate proteins with different biological function, leading to abnormal cell processes, such as proliferation, migration, apoptosis and autophagy. The potential mechanism of microRNA interference might cause targeting of specific mRNA/signalling pathways. Currently, nearly 20 kinds of microRNAs have been demonstrated to participate in the pathogenesis of PAH induced by hypoxia/MCT/Sugen5416 [34, 35]. Additionally, long noncoding RNAs (lncRNAs) are also subject to investigation due to their regulation in cellular and molecular trafficking in process of PAH. LncRNAs possess powerful regulatory functions that affect proliferation, migration and apoptosis in PASMCs, even causing endothelial dysfunction and EndMT [36]. For example, three lncRNAs (Tug1, hoxa cluster antisense RNA3 and maternally expressed gene 3) have been demonstrated to accelerate proliferation and migration of PASMCs via activation of downstream of hypoxia signalling [37–39]. Ultimately, histone modification by acetylation and methylation of specific amino acids influences the development of PAH by regulating gene transcriptional activity and gene expression. Histones, the primary components of chromatin, directly modulate the expression pattern of genes [31]. Existing evidence also shows that histone deacetylase (HDAC) inhibitors might represent promising and emerging therapeutic targets in PAH. Interestingly, the result of Bogaard et al. was not consistent with previous reports with respect to the role of HDAC in PAH [40]. Thus, controversy regarding whether HDAC is “good” or “bad” with respect to PAH still needs further clarification, especially in process of development, treatment, and prognosis of PAH.

### Dysregulation of vascular cells

According to updated knowledge on PAH, pulmonary vascular remodelling is largely caused by excessive PASMC proliferation, HPAEC dysfunction and endothelial to mesenchymal transition. Meanwhile, abnormal migration, apoptosis and autophagy also participate in the progressive development of PAH [41]. Investigations have suggested that BMPR2 and hypoxia-inducible factor (HIF) signalling pathways play a crucial role in the pathogenesis of pulmonary arterial hypertension. The absence of BMPR2 mutations contributes to negative bone morphogenetic protein (BMP) signalling and positive TGF-β signalling, leading to endothelial cell dysfunction, inflammatory cell infiltration, extracellular matrix synthesis and angiogenesis [42, 43]. Therefore, many current studies examining targeted treatment of PAH still focus on the TGF-β pathway. Additionally, increasing evidence indicates that HIF-1α-mediated signalling and mitochondrial metabolism in hypoxia drive the development of cardiovascular disease, including PAH [44]. Elevated HIF-1α induced by hypoxia enhances levels of intracellular Ca^2+^ concentrations in PASMCs by activating extracellular-signal-regulated kinase (ERK)1/2 and p38 mitogen-activated protein kinase (MAPK) pathways to increase transient receptor potential canonical (TRPC) 1 and TRPC6 levels. Furthermore, the aforementioned result might be attributed to activation of Smad1/5/8 in response to BMP-4 [45]. Meanwhile, mitochondrial compensatory reactions produce reactive oxygen species (ROS) under hypoxia, exacerbating the oxygen deficit. ROS also trigger activation of HIF-1 pathways, leading to further oxidative stress injury [46]. In 2015, results of Wright et al. indicated that expression of oestrogen receptor (ER)α is higher in female PASMCs than in male PASMCs from PAH patients, and ERα reverses oestrogen-induced PASMC proliferation by blocking MAPK and Akt signalling pathways [47]. However, of note, hypoxia can only increase expression of ERβ in HPAECs exposed to hypoxia, while ERα expression is not markedly changed. This finding implies that excessive proliferation provoked by hypoxia in PASMCs and HPAECs might mediate differential ER responses and regulate disparate pathways [48]. In addition, IL-33 is regarded as a key inducing cytokine in many lung diseases. Liu et al. [49] found that elevated IL-33 also induced proliferation and adhesion of HPAECs and angiogenesis, contributing to vascular remodelling in hypoxia by binding its receptor ST2 and activating HIF-1α/vascular endothelial growth factor (VEGF) signalling. Currently, evidence in numerous studies suggests that EndMT may lead to pulmonary vascular remodelling. The EndMT process exhibits distinct characteristics, including reduced expression of endothelial proteins and highly increased expression of fibrotic specific genes and extracellular matrix proteins, which induces loss of endothelial function and transformation into a mesenchymal phenotype via the loss of endothelial markers, such as platelet endothelial cell adhesion molecule 1 (PECAM1), and the acquisition of mesenchymal markers, such as smooth muscle actin (α-SMA) [50, 51]. Furthermore, endothelial dysfunction directly causes elevated pulmonary arterial pressure and pulmonary vascular resistance through enhancing the thickness of the vascular intimal and medial layers [28]. Meanwhile, a more recent study revealed that Gremlin-1, a BMP antagonist, triggers EndMT in HPAECs by phosphorylating Smad2/3, but Gremlin-1-mediated EndMT process was reversed by BMP-7 phosphorylation of Smad1/5/8 and inhibition of Smad2/3 phosphorylation [52]. A previous study corroborated the dysfunction of BMP/Smad signalling participating in the development and progression of monocrotaline (MCT)-induced PAH in rats by downregulating p-Smad1 and BMP receptors in PASMCs. Moreover, PASMCs undergoing apoptosis induced by BMP-7 and resistance of BMP-4 to proliferation caused by platelet-derived growth factor (PDGF) were all restrained by impaired BMP/Smad signalling [53]. In addition, it has been widely recognized that an imbalance in vasoactive mediators, such as NO and ET-1, also facilitates vasoconstriction to induce increased pressure and obstruction of pulmonary vessels. Meanwhile, ET-1 also promotes proliferation of PASMCs by inducing the phosphorylation of c-fos and c-jun transcription factors [54]. Overall, accumulating evidence indicates that dysfunction of PASMCs, HPAECs and fibroblasts are all involved in the pathogenesis of PAH. Yet limited data concerning the interaction of those vascular cells has been reported. Accordingly, future studies should emphasize investigation of the interactions among PASMCs, HPAECs and fibroblasts and how they contribute to the development of PAH using in vitro co-culture methods.

**Fig. 2:**
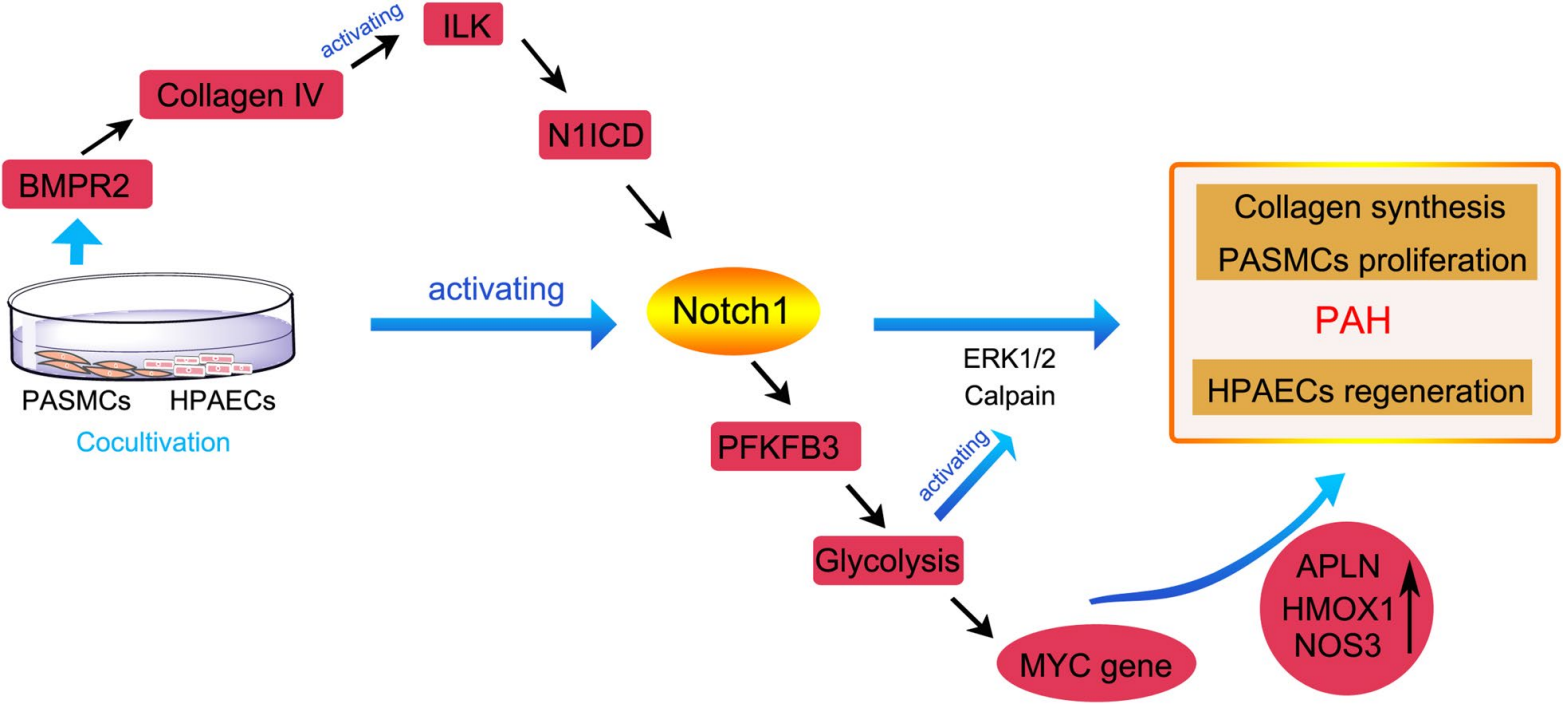
Signalling pathway of glycolysis in PAH. Cocultivation of PASMCs and HPAECs promotes endothelial regeneration and collagen synthesis through BMPR2-Notch1-mediated glycolysis, resulting in the development of PAH. BMPR2 activated Notch1 via mediating the activation of ILK. Meanwhile, *APLN*, *HMOX1* and *NOS3* are *MYC* targets of transcription, those gene of upregulation induced HPAECs proliferation. *PASMCs* pulmonary arterial smooth muscle cells, *HPAECs* human pulmonary arterial endothelial cells, *BMPR2* bone morphogenetic protein receptor 2, *ILK* Integrin-linked kinase, *N1ICD* Notch1 intracellular domain, *PFKFB3* 6-phosphofructo-2-kinase/fructose-2,6-bisphosphatase 3

Metabolic dysfunction in PAH has also been recently reported, for instance, in oestrogen metabolism, mitochondrial metabolism and glucose metabolism. First, oestrogen is primarily catalysed into a metabolite lacking oestrogenic activity called 17β-oestradiol (E2) by cytochrome P450 1B1 (CYP1B1) [55]. Next, E2 is primarily oxidized at C2, C4 and C16 positions, which lead to the generation of metabolites with different biological and oestrogenic activity, including 2-hydroxyestradiol, 4-hydroxyestradiol and 16α-hydroxyestrone [56]. Thus, oestrogen and its metabolites might display physiologically normal or pathogenically deleterious effects on PAH in experiment animals. Furthermore, epidemiological investigations have suggested that morbidity in female PAH patients is higher than in males, indicating that endogenous oestrogen, particularly E2 in female patients, may represent a potential mediator in the pathogenesis of PAH [57]. However, paradoxically, the survival rate of female PAH patients is greater than in male patients [58]. Additionally, abundant evidence has demonstrated that exogenous E2 and its metabolites all possess potent protective effects in established PAH animal models [59]. For example, the non-estrogenic oestradiol metabolite 2-methoxyestradiol restores right heart hypertrophy, aberrant proliferation and inflammatory responses in the lungs of PAH rats induced by MCT [60]. Hence, the “oestrogen paradox” is still considered controversial due to oestradiol and its metabolites exerting both positive and negative effect on PAH [61]. Although significant efforts have been made, understanding the effects endogenous and exogenous oestrogen and how they influence the development, progression and prognosis of PAH still need additional investigation. Second, modern evidence suggests that mitochondrial dysfunction induced by hypoxia also promotes PAH in both in vivo and in vitro experiments, primarily presenting as glucose oxidation phosphorylation towards uncoupled aerobic glycolysis, termed “Warburg metabolism”. During hypoxia, glycolysis is abnormally increased to afford energy for cell survival due to the suppression of mitochondrial respiration. Meanwhile, this abrupt transition also causes excessive proliferation and restrains apoptosis in PASMCs [30, 62]. Further, research from Laszlo et al. verified that the increased glycolysis was driven by upregulated 6-phosphofructo-2-kinase/fructose-2,6-bisphosphatase 3 (PFKFB3) with the function of regulating glycolytic flux in vascular cells. Then, the glycolytic product lactate consequently induces collagen-1 synthesis and PASMC proliferation to facilitate pulmonary vascular remodelling, which occurs due to phosphorylation of ERK1/2 induced by lactate that activates calpain-2, but not calpain-1, at Ser50 [63]. Interestingly, another study validated the presence of elevated glycolysis due to increased expression of PFKFB3. Moreover, elevated glycolysis induced HPAEC regeneration and proliferation by enhancing downstream MYC gene and promoting proliferation and expression of various genes (e.g., APLN, HMOX1, and NOS3). Intriguingly, co-culture of PASMCs and HPAECs unexpectedly caused the activation of Notch homologue 1 (Notch1) by activating BMPR2/integrin-linked kinase (ILK)/Notch1 intracellular domain (N1ICD). Coincidentally, the activated Notch1 mediates glucose metabolism via upregulation of PFKFB3 [64]. Therefore, contact of PASMCs and HPAECs promotes endothelial regeneration through BMPR2-Notch1-mediated glycolysis, as illustrated in Fig. 2. Furthermore, Chen et al. found that increased HIF-1α regulates mitochondrial fission via directly up-regulation of dynamin-related protein 1 (Drp1) expression, which promotes proliferation and inhibits apoptosis in hypoxia-stimulated PASMCs [65].

### Other factors

Experimental and clinical evidence has shown that infiltration of mast cells, macrophages and lymphocytes is largely found in plexiform lesions in PAH patients [66]. Furthermore, proinflammatory cytokines and growth factors, such as tumour necrosis factor (TNF), interleukin (IL)-4, IL-1β, and TGF-β1, secreted by the aforementioned cells contributes to the remodelling of pulmonary vasculature and the recruitment of inflammatory cells [67]. Meanwhile, Banasová et al. also validated that mast cells do indeed participate in the process of pulmonary vascular remodelling by promoting muscularization of peripheral pulmonary arteries, collagen deposition and activating matrix metalloproteinases [68]. Additionally, B and T lymphocytes, dendritic cells, mast cells and neutrophils are also vital initiators of pulmonary vascular remodelling [69]. Interestingly, proinflammatory cytokines not only activate the immune response but also shift T lymphocyte phenotype. For instance, IL-4 drives the shift from macrophages and T cells to activated M2 macrophage and the Th2 phenotype, respectively [70]. In contrast, Th2 associated cytokines also aggravate and amplify the inflammatory response in the pulmonary vessel lesions in PAH [71]. Another study revealed that the early proinflammatory and pro-proliferative responses are drove by the activated complement system through immunoglobulin G (IgG) in hypoxia-induced PAH mice. Moreover, Csf2/GM-CSF was identified as the primary complement-dependent inflammatory mediator. Meanwhile, according to the analysis of network medicine and results of PAH animal experiments, plasma complement signalling might represent a biomarker and/or prognostic factor for clinical outcome in PAH patients [72, 73]. Plentiful biomarkers have been found with respect to PAH diagnostics and prognosis that are involved in the haemodynamics and prognosis of PAH, including IL-6, TNF-α, matrix metalloproteinase (MMP)-9 and CCL-2. Currently, increasing promising inflammatory targets and immunomodulatory pathways have been outlined in an effort to identify effective treatment and prevention of PAH through the results of pre-clinical and clinical research [74]. Hence, great effort should focus on the emerging hotpot IL-6-receptor antagonist, as well as targeting leukotriene B4 and nuclear factor-κB (NF-κB) signalling in different PAH animal models and clinical trials.

**Fig. 3:**
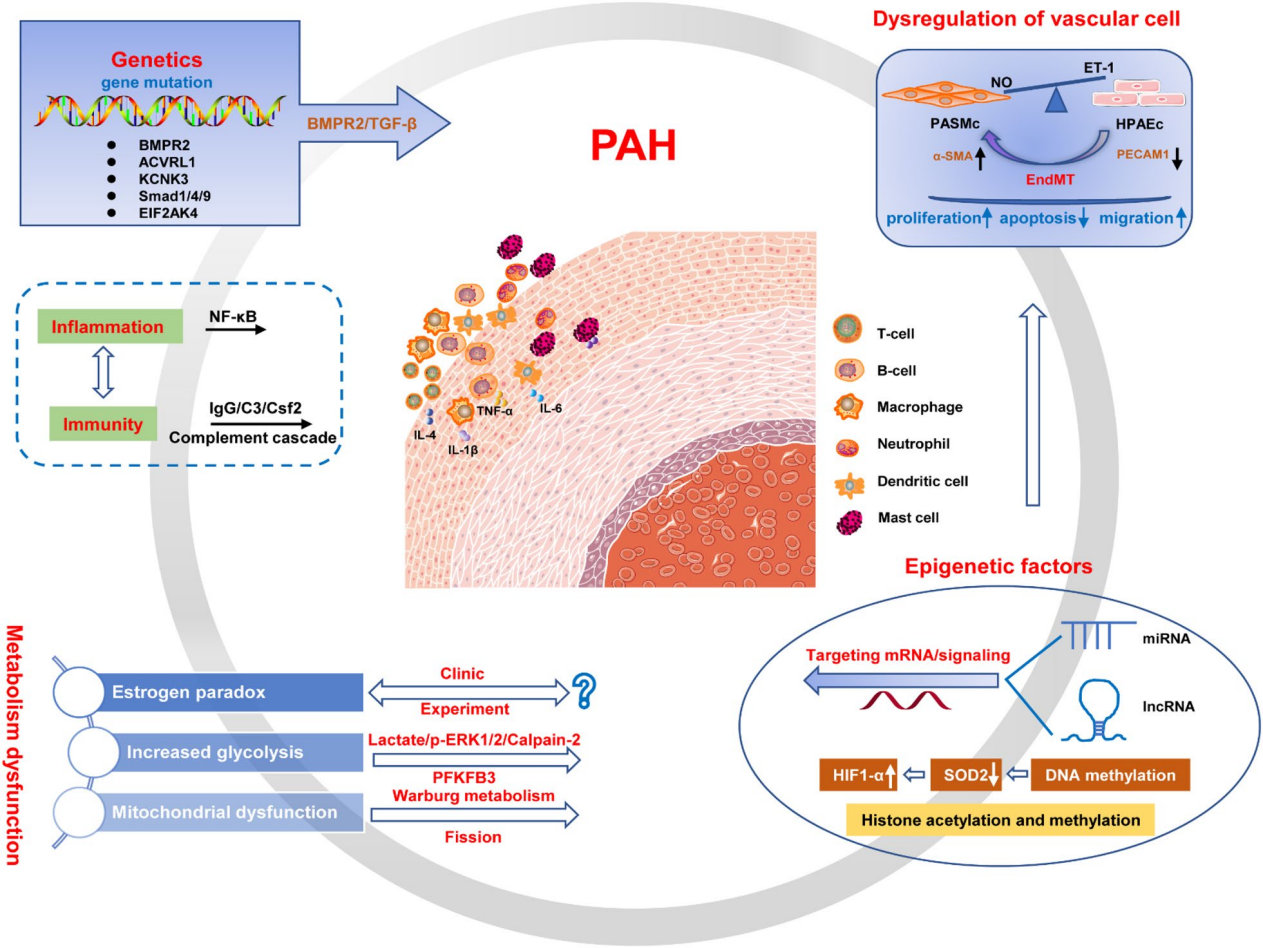
Pathogenesis of PAH in genetic and cellular levels. The main pathogenesis involves in genetic mutations (BMPR2, ACVRL1, KCNK3, Smad1/4/9 and EIF2AK4), dysregulation of vascular cells (HPAECs and PASMc), epigenetic factors (miRNA and lncRNA), metabolic dysfunction (oestrogen, mitochondrial and glucose metabolism), inflammation and immunity (NF-κB and complement cascade). *PAH* pulmonary arterial hypertension, *NO* nitric oxide, *ET-1* endothelin-1, *PASMCs* pulmonary arterial smooth muscle cells, *HPAECs* human pulmonary arterial endothelial cells, *EndMT* endothelial–mesenchymal transition, *PECAM1* platelet endothelial cell adhesion molecule 1, *α-SMA* α-smooth muscle actin, *HIF* hypoxia-inducible factor, *SOD* superoxide dismutase, *PFKFB3* 6-phosphofructo-2-kinase/fructose-2,6-bisphosphatase 3, *IgG* immunoglobulin G

In short, the potential pathogenesis of PAH involves numerous factors due to disease complexity and progression, including genetic mutations, epigenetic factors, dysregulation of PASMCs and HPAECs, metabolic dysfunction, inflammation and immunization (Fig. 3). Importantly, many details remain to be clarified, especially the “oestrogen paradox”.

## Therapy for PAH

### Monomers of natural medicines

**Fig. 4:**
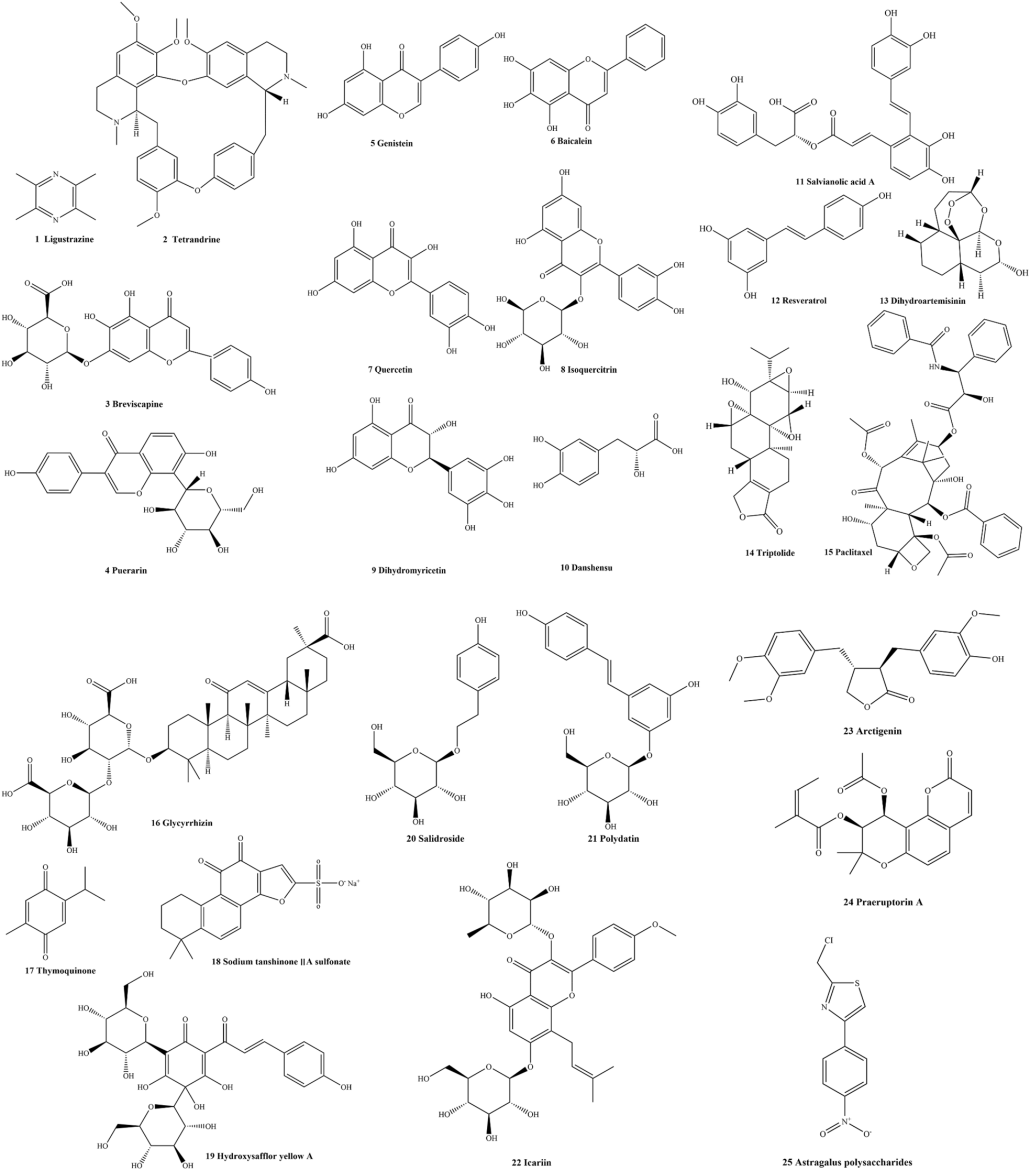
Chemical structures of natural medicines for PAH treatment

**Fig. 5:**
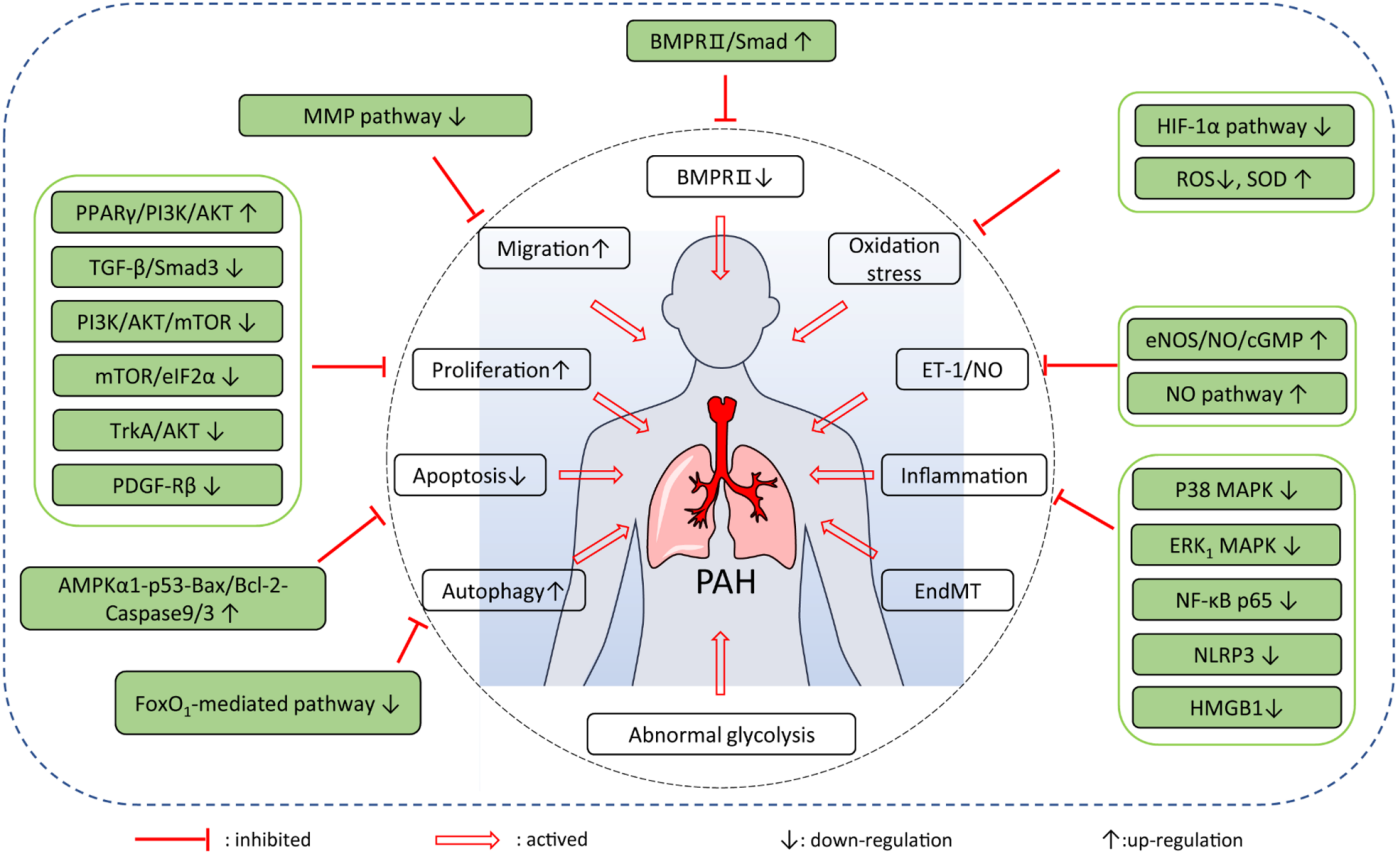
Mechanisms of natural medicines for PAH treatment. The green box represented the modulatory effects of natural medicines for PAH in signalling pathway. The white box indicated that the confirmed pathways, proteins and phenomenon could trigger PAH. For example, the natural medicines downregulated HIF-1α pathway to inhibit oxidation stress response against PAH. Upregulation of BMPR2/Smad by natural medicines could reverse that the low expression of BMPR2 induced development of PAH. Evidently, the NO related pathways influenced the balance of ET-1 and NO for promoting PAH. The inflammation signalling pathways in PAH mainly refered to p38 MAPK, ERK MAPK, NF-κB p65, NLRP3, HMGB1 pathways. The PPARγ/PI3K/AKT, TGF-β-Smad3, PI3K/Akt/mTOR et al. signalling pathways regulated the proliferation of vascular cell in PAH. Moreover, the related signalling pathways of migration, apoptosis, and autophagy also were testified in PAH. Thus, the modulatory effects on specific pathways of natural medicines for PAH were clearly showed in the figure

Multiple natural medicines derived from traditional Chinese medicine have been tested and possess rich pharmacological activities. According to a previous study, approximately 25 types of natural medicines have been found to protect against MCT or hypoxia-induced PAH injury, including alkaloids, flavonoids, polyphenols, glycosides, terpenes, quinones and polysaccharides (Table 2 and Fig. 4). For example, one experiment indicated that ligustrazine may represent a potential therapeutic for rats with PAH in different models (e.g., chronic hypoxia, Sugen5416/hypoxia and monocrotaline-induced PAH) due to the modulation of calcium homeostasis in PASMCs [75]. ET-1 and NO play crucial roles in cardiovascular disease, including PAH. The results demonstrated that levels of vasomotor factors in PAH patients were also notably improved after treatment with ligustrazine injection [76]. The alkaloid tetrandrine derived from a Chinese medicinal herb markedly improved pulmonary artery pressure and right ventricular hypertrophy, possibly through regulating the NO signalling pathway and oxidative stress response in PAH rats induced by monocrotaline. Increased protein kinase type 1 (PKG-1) also directly stimulates endothelial nitric oxide synthase (NOS) to produce NO and inhibit calcium influx, which activates calmodulin. Meanwhile, in vitro experiments demonstrated that tetrandrine exerted antagonistic effects by blocking calcium channels in myocardial cells [77, 78]. A clinical study of breviscapine also verified the above results [79]. Puerarin, a natural flavonoid compound, has been confirmed to clearly ameliorate pulmonary arterial histopathology by inhibiting PASMC proliferation in vitro/in vivo and stalling PASMC cell cycle progression in G1 phase. Furthermore, reduction of cycle-related proteins cyclin A, cyclin E and cyclin D1 was attributed to inhibition of autophagy induced by puerarin administration [80]. Meanwhile, an in vitro experiment demonstrated that puerarin administration reduces ROS and ET-1 levels and increases NO levels in hypoxia-induced HPAECs by activating BMPR2/Smad and peroxisome proliferator-activated receptor-γ (PPARγ)/phosphatidylinositol 3-kinase (PI3K)/Akt signalling pathways [81]. Hypoxia-induced pulmonary vascular remodelling is a critical factor in PAH. Imbalance in ROS and H_2_O_2_ in hypoxia-induced PASMCs was restored by genistein, and the protective effect was thought to involve ER and β-adrenoceptor signalling pathway [82]. In an in vivo experiment, the potential anti-inflammatory activity of baicalein was discovered in MCT-induced PAH rats, resulting in the reduction of TNF-α, IL-1β, IL-6 and the transcriptional regulatory factor NF-κB by regulating NF-κB and BMP/Smad pathways [83, 84]. Another study demonstrated that tyrosine receptor kinase A (TrkA)/AKT signalling was blocked by quercetin, inhibiting PASMCs proliferation and migration, while inducing apoptosis [85]. Meanwhile, its homologue isoquercitrin also exerts a protective effect against PAH induced by MCT, and the potential mechanism might involve attenuation of the PDGF-receptor β signalling pathway [86]. PASMC and HPAEC proliferation, migration, apoptosis and autophagy all participate in the development of PAH. Hence, Li et al. investigated the specific influences of dihydromyricetin in mitigating IL-6 induction in PASMCs, resulting in MMP9 and p-STAT3 being significantly decreased. In other words, dihydromyricetin suppressed PASMCs migration from the middle layer to the inner vessel of pulmonary vessels [87]. Another in vitro study confirmed that danshensu inhibited proliferation of PASMCs, and the protective effect was related to regulation of TGF-β–smad3 pathways [88]. In a similar in vivo model experiment, results suggested that pulmonary haemodynamic abnormalities and pathological morphology were significantly ameliorated by administration of salvianolic acid A. The mechanisms might involve activation of the BMPR2-Smad pathway to reduce ET-1 levels and inhibition of apoptosis due to lung parenchymal damage [89]. Interestingly, the tanshinone IIA derivative of sodium tanshinone IIA sulfonate (STS) attenuated PASMC proliferation induced by hypoxia through suppressing the mammalian target of rapamycin (mTOR)/eukaryotic initiation factor 2α (eIF2α) pathway [90]. Meanwhile, further study found that STS also recovered the deficient BMPR2 in lung of rats with chronic hypoxic PAH and activated downstream p-smad1/5/8 signalling [91]. Additionally, hypoxia-induced PAH might be associated with activation of MAPK/ERK1 and PI3K/AKT signalling, and resveratrol treatment inhibited these pathways, resulting in reduced production of TNF-α, IL-1β, and IL-6 in the lung tissues of rats [92]. The artemisinin derivative of dihydroartemisinin also significantly suppressed cell proliferation, migration, and oxidative stress in hypoxia-induced HPAECs by regulating levels of ROS and SOD [93]. Accumulating evidence has indicated that triptolide possesses a broad spectrum of bioactivities, including alleviating PAH in MCT-induced rats. Moreover, the protective effects of triptolide in PAH may implicate reduced levels of MMP2 and proliferating cell nuclear antigen (PCNA) [94, 95]. At present, the marked anticancer compound paclitaxel also shows notable protective effects in MCT-induced PAH rats by suppressing forkhead box protein O_1_ (FoxO_1_)-mediated autophagy [96]. High mobility group box-1 (HMGB1), a nuclear non-histone DNA-binding protein, regulates the induction of the inflammatory response stimulated by various factors [97]. A study by Yang et al. demonstrated that glycyrrhizin clearly attenuated increased HMGB1 in pulmonary vascular lesions in MCT-induced PAH rats [98]. This observation implies that HMGB1 may represent a novel potential therapeutic target in PAH. In addition, the results indicated that treatment with thymoquinone notably improved pulmonary arterial remodelling by inhibiting related proliferation protein biomarkers PCNA and α-SMA, promoting apoptosis, and downregulating expression of MMP-2. Furthermore, the protective effect of thymoquinone might occur through regulation of the p38 MAPK/NF-κB signalling pathway [99]. The well-known PI3K/Akt/mTOR pathway regulates cell proliferation, differentiation and apoptosis [100]. Pulmonary arterial remodelling is largely evaluated by the right ventricular hypotrophy index (RVHI = [RV/(LV + S)]), and the bioactive constituent hydroxysafflor yellow A, derived from safflower, significantly improved remodelling of pulmonary vasculature and haemodynamic changes by inhibiting RVHI and PCNA [101]. Treatment with 32 mg/kg salidroside significantly reversed the hypoxia-induced right ventricular hypertrophy primarily through upregulating the adenosine A_2a_ receptor (A_2a_R)-related mitochondria-dependent apoptosis pathway. For instance, adenosine monophosphate-activated the protein kinase α-1 (AMPKα1)-p53-Bcl-2-like protein 4 (Bax)/B-cell lymphoma 2 (Bcl-2)-Caspase 9–Caspase-3 pathway. Further study suggested that the therapeutic effect of salidroside was implicated in the inhibition of PASMC proliferation via the AMPKα1-p53–P27/P21 pathway [102, 103]. Additionally, polydatin, characterized by widespread bioactivities, has been found to inhibit proliferation of PASMCs by downregulating PCNA and α-SMA expression [104]. A previous study also verified that icariin extracted from the *Epimedium brevicornum* Maxim improved PAH by MCT in rats through enhancing the NO/cyclic guanosine monophosphate (cGMP) pathway [105]. A large body of evidence has demonstrated that inflammation plays a vital role in the pathogenesis of many diseases, including PAH. The recombinant NLR family pyrin domain containing protein 3 (NLRP3) inflammasome signalling pathway was blocked by administration of arctigenin to resist MCT-induced lung injury in rats [106]. In s chronic hypoxic environment, HIF-1 increases Ca^2+^ influx and TRPC expression in PASMCs [107]. Interestingly, elevated levels of Ca^2+^ and store-operated Ca^2+^ channels (SOCCs) were significantly inhibited by praeruptorin A administration [108]. Inflammation also plays a vital role in the development of PAH. For instance, astragalus polysaccharides decrease levels of the inflammatory cytokine TNF-α, IL-1β and IL-6 to resist pulmonary artery hypertension induced by MCT. Meanwhile, endothelial nitric oxide synthase (eNOS)/NO signalling pathways were also activated by astragalus polysaccharides [109]. Overall, natural medicines act by primarily inhibiting pulmonary arterial remodelling through different mechanisms, including inhibiting PASMC proliferation, promoting PASMC apoptosis, regulating vasomotor factors, restraining oxidative stress, alleviating the inflammatory response and suppressing autophagy (Table 3 and Fig. 5).

**Table 2:** Chemical constituents of natural medicines to treat PAH

Classification	No	Chemical component	Molecular formula	IUPAC name	References
Alkaloids	1	Ligustrazine	C_8_H_12_N_2_	2,3,5,6-tetramethylpyrazine	[75, 76]
Alkaloids	2	Tetrandrine	C_38_H_42_N_2_O_6_	(1*S*,14*S*)-9,20,21,25-tetramethoxy-15,30-dimethyl-7,23-dioxa-15,30-diazaheptacyclo[22.6.2.2^3,6^.1^8,12^.1^14,18^.0^27,31^.0^22,33^]hexatriaconta-3(36),4,6(35),8,10,12(34),18,20,22(33),24,26,31-dodecaene	[77]
Flavonoids	3	Breviscapine	C_21_H_18_O_12_	(2*S*,3*S*,4*S*,5*R*,6*S*)-6-[5,6-dihydroxy-2-(4-hydroxyphenyl)-4-oxochromen-7-yl]oxy-3,4,5-trihydroxyoxane-2-carboxylic acid	[79]
Flavonoids	4	Puerarin	C_21_H_20_O_9_	7-hydroxy-3-(4-hydroxyphenyl)-8-[(2*S*,3*R*,4*R*,5*S*,6*R*)-3,4,5-trihydroxy-6-(hydroxymethyl) oxan-2-yl] chromen-4-one	[80, 81]
Flavonoids	5	Genistein	C_15_H_10_O_5_	5,7-dihydroxy-3-(4-hydroxyphenyl) chromen-4-one	[82]
Flavonoids	6	Baicalein	C_15_H_10_O_5_	5,6,7-trihydroxy-2-phenylchromen-4-one	[83, 84]
Flavonoids	7	Quercetin	C_15_H_10_O_7_	2-(3,4-dihydroxyphenyl)-3,5,7-trihydroxychromen-4-one	[85]
Flavonoids	8	Isoquercitrin	C_21_H_20_O_12_	2-(3,4-dihydroxyphenyl)-5,7-dihydroxy-3-[(2*S*,3*R*,4*S*,5*S*,6*R*)-3,4,5-trihydroxy-6-(hydroxymethyl) oxan-2-yl] oxychromen-4-one	[86]
Flavonoids	9	Dihydromyricetin	C_15_H_12_O_8_	(2*R*,3*R*)-3,5,7-trihydroxy-2-(3,4,5-trihydroxyphenyl)-2,3-dihydrochromen-4-one	[87]
Polyphenols	10	Danshensu	C_9_H_10_O_5_	(2*R*)-3-(3,4-dihydroxyphenyl)-2-hydroxypropanoic acid	[88]
Polyphenols	11	Salvianolic acid A	C_26_H_22_O_10_	(2*R*)-3-(3,4-dihydroxyphenyl)-2-[(*E*)-3-[2-[(*E*)-2-(3,4-dihydroxyphenyl)ethenyl]-3,4-dihydroxyphenyl]prop-2-enoyl]oxypropanoic acid	[89]
Polyphenols	12	Resveratrol	C_14_H_12_O_3_	5-[(*E*)-2-(4-hydroxyphenyl)ethenyl]benzene-1,3-diol	[92]
Terpenes	13	Dihydroartemisinin	C_15_H_24_O_5_	(1*R*,4*S*,5*R*,8*S*,9*R*,10*S*,12*R*,13*R*)-1,5,9-trimethyl-11,14,15,16-tetraoxatetracyclo[10.3.1.0^4,13^.0^8,13^] hexadecan-10-ol	[93]
Terpenes	14	Triptolide	C_20_H_24_O_6_	(1*S*,2*S*,4*S*,5*S*,7*R*,8*R*,9*S*,11*S*,13*S*)-8-hydroxy-1-methyl-7-propan-2-yl-3,6,10,16-tetraoxaheptacyclo[11.7.0.0^2,4^.0^2,9^.0^5,7^.0^9,11^.0^14,18^]icos-14(18)-en-17-one	[94, 95]
Terpenes	15	Paclitaxel	C_47_H_51_NO_14_	[(1*S*,2*S*,3*R*,4*S*,7*R*,9*S*,10*S*,12*R*,15*S*)-4,12-diacetyloxy-15-[(2*R*,3*S*)-3-benzamido-2-hydroxy-3-phenylpropanoyl]oxy-1,9-dihydroxy-10,14,17,17-tetramethyl-11-oxo-6-oxatetracyclo[11.3.1.0^3,10^.0^4,7^]heptadec-13-en-2-yl] benzoate	[96]
Terpenes	16	Glycyrrhizin	C_42_H_62_O_16_	(2*S*,3*S*,4*S*,5*R*,6*R*)-6-[(2*S*,3*R*,4*S*,5*S*,6*S*)-2-[[(3*S*,4*aR*,6*aR*,6*bS*,8*aS*,11*S*,12*aR*,14*aR*,14*bS*)-11-carboxy-4,4,6*a*,6*b*,8*a*,11,14*b*-heptamethyl-14-oxo-2,3,4*a*,5,6,7,8,9,10,12,12*a*,14*a*-dodecahydro-1*H*-picen-3-yl]oxy]-6-carboxy-4,5-dihydroxyoxan-3-yl]oxy-3,4,5-trihydroxyoxane-2-carboxylic acid	[98]
Quinones	17	Thymoquinone	C_10_H_12_O_2_	2-methyl-5-propan-2-ylcyclohexa-2,5-diene-1,4-dione	[99]
Quinones	18	sodium tanshinone IIA sulfonate (STS)	C_19_H_17_NaO_6_S	sodium;1,6,6-trimethyl-10,11-dioxo-8,9-dihydro-7*H*-naphtho[1,2-g][1]benzofuran-2-sulfonate	[90, 91]
Quinones	19	Hydroxysafflor yellow A	C_27_H_32_O_16_	2,5-dihydroxy-6-[(*E*)-1-hydroxy-3-(4-hydroxyphenyl)prop-2-enylidene]-2,4-bis[(2*S*,3*R*,4*R*,5*S*,6*R*)-3,4,5-trihydroxy-6-(hydroxymethyl)oxan-2-yl]cyclohex-4-ene-1,3-dione	[101]
Glycosides	20	Salidroside	C_14_H_20_O_7_	(2*R*,3*S*,4*S*,5*R*,6*R*)-2-(hydroxymethyl)-6-[2-(4-hydroxyphenyl)ethoxy]oxane-3,4,5-triol	[102, 103]
Glycosides	21	Polydatin	C_20_H_22_O_8_	(2*S*,3*R*,4*S*,5*S*,6*R*)-2-[3-hydroxy-5-[(*E*)-2-(4-hydroxyphenyl)ethenyl]phenoxy]-6-(hydroxymethyl)oxane-3,4,5-triol	[104]
Glycosides	22	Icariin	C_33_H_40_O_15_	5-hydroxy-2-(4-methoxyphenyl)-8-(3-methylbut-2-enyl)-7-[(2*S*,3*R*,4*S*,5*S*,6*R*)-3,4,5-trihydroxy-6-(hydroxymethyl)oxan-2-yl]oxy-3-[(2*S*,3*R*,4*R*,5*R*,6*S*)-3,4,5-trihydroxy-6-methyloxan-2-yl]oxychromen-4-one	[105]
Lignans	23	Arctigenin	C_21_H_24_O_6_	(3*R*,4*R*)-4-[(3,4-dimethoxyphenyl)methyl]-3-[(4-hydroxy-3-methoxyphenyl)methyl]oxolan-2-one	[106]
Coumarins	24	Praeruptorin A	C_21_H_22_O_7_	[(9*S*,10*S*)-10-acetyloxy-8,8-dimethyl-2-oxo-9,10-dihydropyrano[2,3-f]chromen-9-yl] (*Z*)-2-methylbut-2-enoate	[108]
Polysaccharides	25	Astragalus polysaccharides	C_10_H_7_ClN_2_O_2_S	2-(chloromethyl)-4-(4-nitrophenyl)-1,3-thiazole	[109]

**Table 3:** Pharmacological effects of monomers derived from natural medicines for PAH

Natural medicines	Efficacy	Cell lines/animals	Dose/concentration	Mechanisms of action	Refs
Ligustrazine	Regulating calcium homeostasis	SD rats	100 mg/kg (p.o.)	HIF-1α↓, basal [Ca^2+^]_i_↓, SOCE↓, TRPC1↓, TRPC6↓	[75]
	Regulating vasomotor factors	PAH patient	120 mg/day (i.v.)	ET-1↓, NO↑	[76]
Tetrandrine	Inhibiting oxidative stress	SD rats	50 mg/kg (i.p.)	iNOS↓, PKG-1↑, SOD↑, MDA↓	[77, 78]
Breviscapine	Regulating vasomotor factors	PAH patient	50 mg/day (i.v.)	ET-1↓, NO↑	[79]
Puerarin	Inhibiting PASMC proliferation	SD rats	80 mg/kg (p.o.)	LC3B-II↓, BECN-1↓, ATG5↓, SQSTM1↑	[80]
	Inhibiting oxidative stress	HPAECs	30 µmol/L	BMPR2/Smad↑, PPARγ/PI3K/Akt↑	[81]
Genistein	Inhibiting oxidative stress	PASMCs	50 µmol/L	ROS↓, SOD↑, H_2_O_2_↑	[82]
Baicalein	Inhibiting inflammatory response	SD rats	100 mg/kg (p.o.)	TNF-α↓, IL-1β↓, IL-6↓	[83]
	Inhibiting inflammatory response	Wistar rats	100 mg/kg (p.o.)	NF-κB p65↓, BMPR2↑, BMP-4↑, BMP-9↑, Smad1/5/8↑	[84]
Quercetin	Inhibiting PASMC proliferation	PASMCs	60 µmol/L	MMP2↓, MMP9↓, Bax/Bcl-2↑; Cyclin B1↓	[85]
Isoquercitrin	Inhibiting PASMC proliferation	PASMCs	30 µmol/L	PCNA↓, α-SMA↓, Cyclin D1↓, CDK4↓, p-PDGF-Rβ↓	[86]
Dihydromyricetin	Inhibiting PASMC migration	PASMCs	100 mg/kg	MMP9↓, p-STAT3↓	[87]
Danshensu	Inhibiting PASMC proliferation	PASMCs	30 µg/mL	Regulating TGF-β-smad3 pathway	[88]
Salvianolic acid A	Improving pulmonary vascular remodelling	SD rats	3 mg/kg (p.o.)	AST↓, ALT↓, NT-proBNP↓, RVSP↓, ET-1↓, BMPR2↑, Smad1/5↑	[89]
STS	Inhibiting PASMC proliferation	PASMCs	10 ng/mL	mTOR↓, eIF2α↓, c-myc↓	[90]
	Activating BMPR2 signalling pathway	SD rats	30 mg/kg (i.p.)	BMPR2↑, CAV1↑, p-smad1/5/8↑	[91]
Resveratrol	Inhibiting inflammatory response	SD rats	40 mg/kg (p.o.)	TNF-α↓, IL-1β↓, IL-6↓	[92]
Dihydroartemisinin	Inhibiting HPAEC proliferation	HPAECs	60 µmol/L	ROS↓, NO↑, SOD↑	[93]
Triptolide	Inhibiting MMP pathways	SD rats	0.25 mg/kg (i.p.)	MMP2↓, MMP9↓	[94]
	Inhibiting PASMC proliferation	SD rats	0.25 mg/kg (i.p.)	PCNA↓, caspase-3↑	[95]
Paclitaxel	Inhibiting autophagy	SD rats	5 mg/kg (i.v.)	p-FoxO_1_↓, RVSP↓, LC3A↓, LC3B↓	[96]
Glycyrrhizin	Inhibiting inflammatory response	SD rats	50 mg/kg (i.p.)	HMGB1↓, survival rate↑, ET-1↓	[98]
Thymoquinone	Inhibiting pulmonary arterial remodelling	SD rats	16 mg/kg (p.o.)	PCNA↓, α-SMA↓, MMP2↓, Bax/Bcl-2↑, cleaved caspase-3↑	[99]
Hydroxysafflor yellow A	Inhibiting PASMC proliferation	Wistar rats	10 mg/kg (i.p.)	RVHI↓, PCNA↓	[101]
Salidroside	Promoting apoptosis	BALB/C mice	32 mg/kg (p.o.)	Bax/Bcl-2↑, caspase 9↑, cleaved caspase-3↑, A_2a_R↑	[102]
	Inhibiting PASMC proliferation	PASMCs	500 µmol/L	AMPKα1↑, P53↑, P27↓, P21↓, PCNA↓, caspase-3↑	[103]
Polydatin	Inhibiting PASMC proliferation	PASMCs	100 ng/mL	PCNA↓, α-SMA↓	[104]
Icariin	Regulating vasomotor factors	SD rats	40 mg/kg (p.o.)	NO↑, eNOS↑, cGMP↑, PDE5↓	[105]
Arctigenin	Inhibiting inflammatory response	SD rats	50 mg/kg (i.p.)	NLRP3↓, IL-1β↓	[106]
Praeruptorin A	Inhibiting PASMC proliferation	PASMCs	20 µmol/L	Basal Ca^2+^↓, SOCE↓	[108]
Astragalus polysaccharides	Inhibiting inflammatory response	SD rats	200 mg/kg	eNOS↑, NO↑, TNF-α↓, IL-1β↓, IL-6↓	[109]

### Extracts of natural medicines

Traditional Chinese medicines have been verified to possess extensive pharmacological activities, e.g. anticancer effect, antioxidant effect, neuroprotective effect, anti-inflammatory effect and anti-COVID-19 effect [110–112]. Plenty of evidence had indicated that the herbal medicines such as *Angelica sinensis*, *Astragalus membranaceus, Rhodiola crenulata* and *Polygonum cuspidatum* exerted potential protective effects on PAH in vivo and in vitro experiments [113]. For instance, evidence had indicated the water extract of *Rhodiola crenulata* could improve PAH via suppressing the fatty acid oxidation and autophagy in MCT-induced rats, and the acylcarnitine might be regarded as a potential therapeutic target in PAH [114]. The ethyl acetate extract of *Sceptridium* ternatum was found to downregulate the α-SMA and NF-κB expression of pulmonary artery in MCT-induced PAH rats. Thus, the ethyl acetate extract of *Sceptridium* ternatum could decrease the mPAP and RVHI by improving the pulmonary vascular remodelling [115]. Blueberry, a fruit with rich amounts of antioxidant, had been proved that the identified total phenolics, flavonoids and anthocyanidins in blueberry exetract significantly inhibited the activity of nicotinamide adenine dinucleotide phosphate (NADPH) and the expression of endothelin receptor A (ETA) to prevent PAH [116]. That implied that the natural antioxidants derived from natural product possibly deemed to be a novel treatment strategy in PAH. Additionally, results of in vivo and in vitro experiments showed that the water extract of *Moringa oleifera leaf* could increase the production of NO mediated by endothelial cells to lower the mPAP in rats. And the in vitro experiment indicated that the protective effect of the water extract of *Moringa oleifera leaf* might owe to the activation of eNOS/NO/cGMP signalling pathway [117]. Another study demonstrated that the water extract of *Terminalia arjuna* could reduce the mPAP and RVHI against MCT-induced PAH rats via inhibiting oxidative stress and apoptosis pathway [118]. Previous study pointed out that the intervention of water extract of barberry (*Berberis integerrima*) also prevented PAH through improving mPAP and RVSP in PAH rats, yet the levels of ET-1 and glutathione peroxidase had not significantly changed compared with the model group. That means that the potential mechanisms of barberry for the treatment of PAH still need to further clarify [119]. The methanol and water extract of *Withania somnifera* was testified to decrease the elevated RVSP and RVHI in PAH rats induced by MCT. Furthermore, the expression levels of PCNA, ROS, TNF-α, NF-κB and HIF-1α were all notably down-regulated after the treatment of *Withania somnifera* extract, which showed that its protective effects might be related to the inflammation, oxidative stress and HIF-1α signalling pathway. Besides, the main active ingredients in the extract of *Withania somnifera* was identified as the withaferin A [120]. Thus, the withaferin A was likely to be the main responsible for the specific beneficial effects of *Withania somnifera* extract. In addition, the hydromethanolic extract of *Mimosa pigra* also possessed the positive effects for PAH by alleviating the abnormal mPAP and RVHI in PAH rats, and the in vitro experiments revealed that the extract of *Withania somnifera* had strong antioxidant activity through regulating activity of eNOS [121]. In short, increasing evidences demonstrate that plenty of natural medicines show favourable bioactivities for PAH, especially the anti-oxidative and anti-inflammatory effects. As shown in Table 4, major experiments mainly focus on the animal model rather than the cell model. Thus, the in-depth cellular and molecular level of study still urgently need to be executed in next research. Besides, those extracts still contain abundant potential active compounds need to be investigated by in vivo and in vitro experiments.

**Table 4:** Pharmacological effects of extracts derived from natural medicines for PAH

Chinese herbs	Extractive fraction	Models	Dose/concentration	Efficacy	Refs
*Rhodiola crenulata*	Water extract	SD rats	5 g/kg (p.o.)	Decadienyl-l-carnitine↓, CPT1A↓, PPARγ↓, LC3B↓, ATG7↓	[114]
*Sceptridium* ternatum	Ethyl acetate extract	SD rats	5 g/kg (p.o.)	mPAP↓, RVHI↓, α-SMA↓, NF-κB↓	[115]
*Blueberry*	Blueberry extract	Wistar rats	100 mg/kg (p.o.)	mPAP↓, SOD↑, ETA↓, NADPH↓	[116]
*Moringa oleifera leaf*	Water extract	Wistar rats	30 mg/kg (i.v.)	mPAP↓, NO↑	[117]
*Terminalia arjuna*	Water extract	Wistar rats	250 mg/kg (p.o.)	mPAP↓, RVHI↓, Bcl2/Bax↑, NADPH↓	[118]
*Barberry*	Water extract	Wistar rats	200 mg/kg (p.o.)	RVSP↓, mPAP↓	[119]
*Withania somnifera*	Methanol and water extract	SD rats	100 mg/kg (p.o.)	RVSP↓, RVHI↓, PCNA↓, ROS↓, TNF-α↓, NF-κB↓, HIF-1α↓	[120]
*Mimosa pigra*	Hydromethanolic extract	Wistar rats	400 mg/kg (p.o.)	mPAP↓, RVHI↓, p38 MAPK↓,	[121]

### MicroRNA regulation

**Fig. 6:**
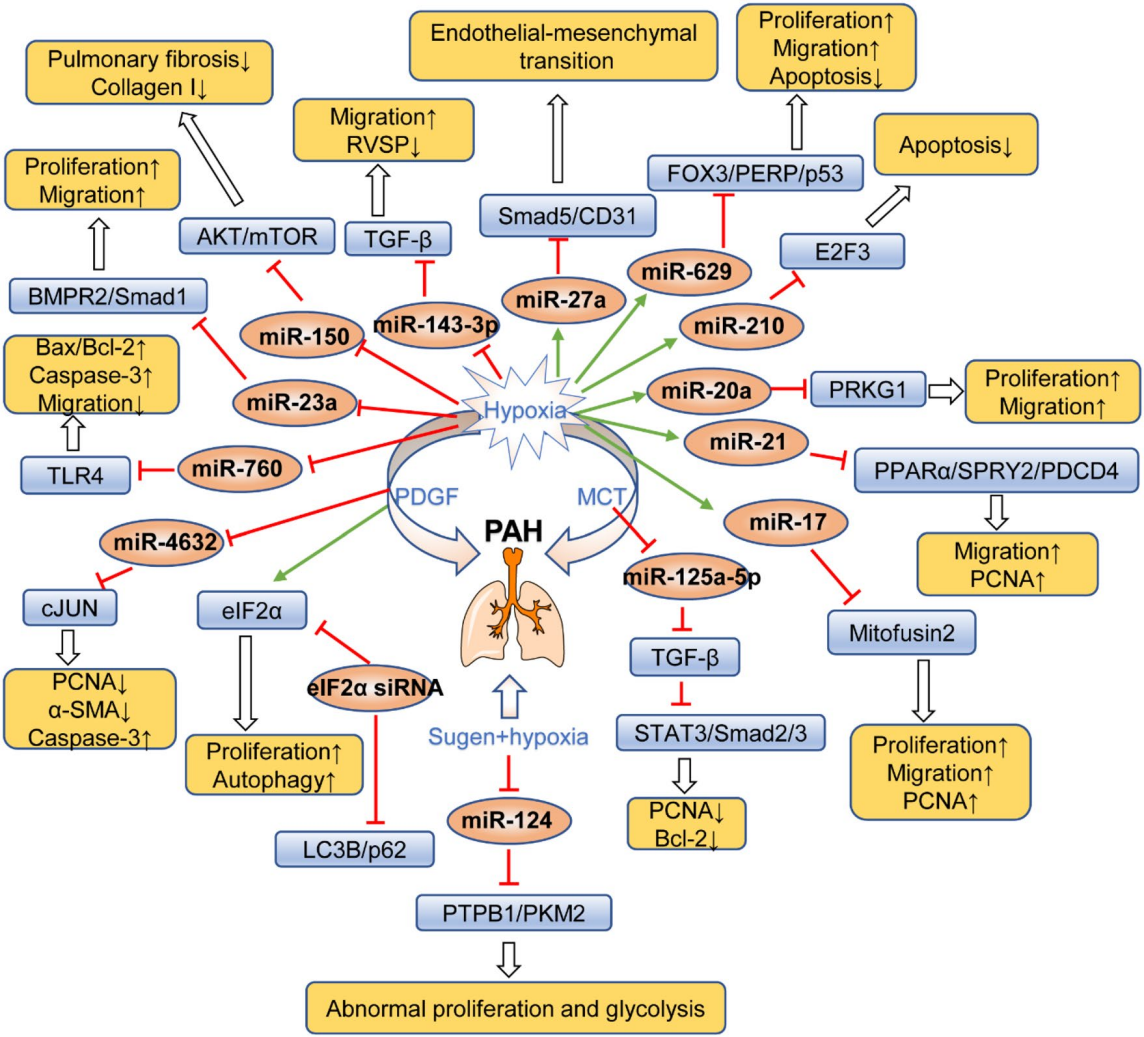
Regulatory mechanisms of microRNAs in PAH. The green arrow indicated the promoted effects of hypoxia or PDGF on microRNAs, yet the red T-shape showed the inhibited effects. The blue box represented the targets or signalling pathways of microRNAs in PAH. The yellow box showed the beneficial effects of microRNAs for the treatment of PAH. *PDGF* platelet-derived growth factor, *MCT* monocrotaline, *PCNA* proliferating cell nuclear antigen, *TGF-β* transforming growth factor-β, *BMPR2* bone morphogenetic protein receptor 2, *eIF2α* eukaryotic initiation factor 2α, *TLR4* toll-like receptor 4, *RVSP* right ventricular systolic pressure, *FOXO3* forkhead box O3, *PERP* p53 apoptosis effector related to PMP‐22, *PPARα* peroxisome proliferator-activated receptor-α, *PDCD4* programmed cell death protein 4, *SPRY2* Sprouty 2, *PTBP1* polypyrimidine tract binding protein, *PKM2* pyruvate kinase M2

MicroRNAs (miRNAs), small non‐coding RNAs 21–23 nucleotides in length, have been reported to participate in many biological processes (e.g., cell proliferation, migration, invasion and apoptosis) [122]. For instance, microRNA-629 promotes PASMC proliferation and migration, suppresses apoptosis by downregulating the target of forkhead box O3 (FOXO3) and p53 apoptosis effector related to PMP‐22 (PERP) in hypoxia-induced PASMCs [123]. Another report also showed that overexpression of microRNA-150 relieved pulmonary fibrosis and collagen I in hypoxia-induced rats, and the specific mechanism is thought to occur through inhibition of the AKT/mTOR signalling pathway [124]. Additionally, one study reported that eukaryotic initiation factor 2α (eIF2α) might represent a vital proliferation protein in PDGF-evoked PASMCs. Moreover, pulmonary vascular remodelling was inhibited by eIF2α siRNA [125]. BMPR2 gene mutations leading to a series of pulmonary changes have been recognized as important mediators of pathogenesis during the development of PAH. Interestingly, microRNA-23a directly targets BMPR/Smad1 to promote hypoxia-induced PASMC proliferation and migration [126]. In addition, microRNA-760 also promotes PASMC apoptosis and restrains migration through downregulating toll-like receptor 4 (TLR4) [127]. Similarly, results also revealed that overexpression of microRNA-17 mediated hypoxia-induced PASMC proliferation and apoptosis by decreasing mitofusin 2 expression [128]. The PAH targets programmed cell death protein 4 (PDCD4), sprouty 2 (SPRY2) and peroxisome proliferator-activated receptor-α (PPARα) are regulated by microRNA-21 in hypoxia-triggered PASMCs. Furthermore, microRNA-21 directly targets PPARα, which was verified by 3′-untranslated region luciferase-based reporter gene assays [129]. Investigation demonstrated that hypoxia increases the expression of microRNA-21a, resulting in PASMCs proliferation and migration by targeting of protein kinase cGMP-dependent type I (PRKG1) [130]. In addition, microRNA-210 and microRNA-4632 both exert clear regulatory effects on PASMC apoptosis by targeting the transcription factor E2F3 and c-Jun, respectively [131, 132]. In a study examining MCT-induced PAH rats, the results revealed that pulmonary vascular remodelling was significantly improved in response to microRNA-125a-5p via negative regulation of downstream Smad2/3 and STAT3 signalling [133]. Abnormal glycolysis is also a typical characteristic of PAH, and one study suggested that decreased microRNA-124 cause dysregulation of glycolysis by elevating the expression of polypyrimidine tract binding protein (PTBP1) and pyruvate kinase M2 (PKM2) in Sugen5416 and hypoxia-induced PAH rats [134]. Additionally, right ventricular hypertrophy (RVH) in hypoxia-induced mice was significantly ameliorated by reduced microRNA-143-3p expression. Abundant signalling pathways involved in the pathogenesis of PAH regulate the expression of miR-143 by activating its promoter region [135]. Meanwhile, downregulation of microRNA-27a increased levels of p-Smad5 and CD31, reducing α-SMA and vimentin expression, which exhibited protect effects against EndMT-induced pulmonary vascular remodelling. Subsequently, elevated p-Smad5 upregulated Id2 levels, and cascade reactions contributed to reducing the levels of snail and twist [136, 137]. As shown in Table 5 and Fig. 6, microRNAs active specific targets to regulate related signalling pathways involved in the proliferation, migration, apoptosis and glycolysis of PASMCs in response to distinct stimuli.

**Table 5:** Regulatory effects of microRNAs in PAH

MicroRNAs	Environment	Target	Function	Refs
MicroRNA-629	Hypoxia-induced PASMCs	FOXO3↓, PERP↓	PASMCs proliferation↑, migration↑, cell apoptosis↓	[123]
MicroRNA-150	Hypoxia-induced rats	AKT/mTOR↓	Cardiac output↓, pulmonary fibrosis↓, collagen fibre↓, collagen I↓, α-SMA↓, TGF-β1↓	[124]
eIF2α siRNA	PDGF-induced PASMCs	eIF2α↓, LC3B↓	PASMCs proliferation↓, p62↑, autophagy↓	[125]
MicroRNA-23a	Hypoxia-induced PASMCs	BMPR2↓, Smad1↓	PASMCs proliferation↑, migration↑, PCNA↑, P-smad1↓	[126]
MicroRNA-760	Hypoxia-induced PASMCs	TLR4↓	Caspase-3↑, Bax/Bcl-2↑, migration↓	[127]
MicroRNA-17	Hypoxia-induced PASMCs	Mitofusin 2↓	PCNA↑, cleaved caspase-3↓	[128]
MicroRNA-21	Hypoxia-induced PASMCs	PPARα↓, PDCD4↓	PCNA↑, SPRY2↓, migration↑	[129]
MicroRNA‐20a	Hypoxia-induced PASMCs	PRKG1↓	Proliferation↑, migration↑, PKG↓, α-SMA↑	[130]
MicroRNA-210	Hypoxia-induced PASMCs	E2F3↓	Apoptosis↓	[131]
MicroRNA-4632	PDGF-induced PASMCs	c-Jun↓	Apoptosis↑, proliferation↓	[132]
MicroRNA-125a-5p	MCT-induced PAH rats	TGF-β1↓, STAT3↓	IL-6↓, Smad2/3↓, PCNA↓, Bcl-2↓	[133]
MicroRNA-124	Sugen-hypoxia-induced rats	PTBP1↓, PKM2↓	BMPR2↑, restoring glycolytic	[134]
MicroRNA-143-3p	Hypoxia-induced mice	TGF-β↓	Migration↓, RVSP↓, RVH↓	[135]
MicroRNA-27a	Hypoxia-induced rats	Smad5↑	Vimentin↓, CD31↑, α-SMA↓, p-Smad5↑,	[136]

### Other therapies

Beyond treatments already discussed, emerging promising therapies for PAH have also attracted increasing interest, such as stem cell (SC) therapy, T helper cell homeostasis and the gut microbiota. SCs are multipotent progenitor cells, especially mesenchymal SCs, possessing strong bioactive abilities, including the ability to differentiate into PASMCs [138]. A recent meta-analysis of results suggested that SC therapy significantly improves mPAP and RVSP in PAH animals, indicating that SC treatment for PAH has promising prospects, particularly induced pluripotent stem cells. Unfortunately, the detailed dose–effect of SCs and their efficacy for PAH requires further study to provide standardization for clinical trials [139]. Moreover, increasing research has implied that imbalance in the immune response also participates in the pathogenesis of PAH. Therefore, Li et al. investigated the causes of T-cell homeostasis in PAH rats induced by hypoxia. The results indicated that imbalance in the ratio of Th17/Treg cells was recovered by the repressive Ras homologue family member A-Rho kinase (RhoA–ROCK) pathway by modulating phosphorylation of STAT3/STAT5 [140]. Hence, further research should be performed to identify effective medicines for the treatment of PAH by targeting the RhoA–ROCK pathway. In addition, dysbiosis of gut microbiota also influences immune regulation in PAH due to initiating early perivascular inflammation caused by changes in the gut microbiota’s composition and function [141]. Furthermore, another study found that deficiency of vitamin D and iron caused by gut dysbiosis accelerated the progression of PAH animal models. Fortunately, the deteriorated progression of PAH was attenuated by flavonoids, such as quercetin [142]. Meanwhile, pulmonary vascular remodelling was characterized by abnormal hypertrophy in adventitial, medial and intimal arteries, caused by excessive proliferation and inhibition of apoptosis in vascular cells in the middle and inner pulmonary vasculature [143]. Therefore, elucidating how to enhance apoptosis of PASMCs and HPAECs is a novel strategy for reversing pulmonary vascular remodelling. For instance, increasing new potential targets have focused on Rho kinase, voltage-gated potassium channels, apoptosis signal-regulating kinase 1 and the bradykinin receptor [144].

## Clinical trials for PAH

At present, the majority of clinical studies primarily concentrate on vasodilators, phosphodiesterase type 5 inhibitors and endothelin receptor antagonist analogues (e.g., prostacyclin, udenafil and ambrisentan) [145–147]. However, few clinical studies of natural medicine for the management of PAH were found. The monomer tetramethylpyrazine (TMP), derived from the traditional Chinese medicine *Ligusticum chuanxiong* Hort., was shown to restrain pulmonary hypertension in hypoxia-, MCT- and Sugen5416/hypoxia-induced PAH rats, respectively. Furthermore, the mechanism was attributed to inhibition of intracellular calcium homeostasis in PASMCs after administration of 100 mg/kg/day TMP. Meanwhile, a small cohort in a clinical trial also validated that TMP (100 mg, t.i.d. for 16 weeks) significantly improved patients with PAH/CTEPH by increasing the 6-min walk distance (6MWD) and improving 1-min heart rate recovery [75]. Unfortunately, given the small sample size and single-blinded nature of the clinical study, these results are insufficient to support the efficacy and safety of TMP for the treatment of PAH. Meanwhile, numerous differential end-points, such as traditional pulmonary haemodynamics of mPAP and PVR, give rise to limitations; therefore, reasonable outcomes in clinical trials of PAH should be formulated to preferentially assess the trial efficiency according to the disease’s biological characteristic and biomarkers. For example, the outcome time to clinical worsening (TTCW) has been widely accepted as the primary end-point for PAH in clinical research. However, the relationship between TTCW and progression, survival, and even mortality, in PAH is not currently standardized. Based on the clinical importance and frequency of end-points in PAH, a corresponding weighting coefficient should be established to promote improved clinical trial design [148, 149]. In addition, the pharmacokinetics and pharmacodynamics of the potential natural medicines under investigation should be emphasized to support their efficacy in subsequent clinical study. Rigorous randomized controlled, double blind and multicentre clinical studies are still needed to build upon previous knowledge.

## Conclusions and perspective

We comprehensively outlined the current status of PAH in pathology, pathogenesis, therapeutic natural medicine and their mechanisms, and clinical trials. First, results indicate that pulmonary vascular remodelling, a typical characteristic of PAH, primarily causes extracellular matrix deposition, endothelial injury and EndMT, accompanied by uncontrolled proliferation and inhibition of apoptosis in middle and inner pulmonary vessels of PASMCs and HPAECs. Therefore, PAH patients ultimately present with elevated mPAP and increased right ventricular systolic pressure due to vascular stenosis and occlusion of pulmonary vasculature. Second, it is well known that heritable PAH generally exhibits mutations in the BMPR2 gene, as well newly identified ACVRL1, ENG, SMAD1, KCNK3 and EIF2AK4. Unfortunately, deficits in these genes also influence proliferation, migration and apoptosis of vascular cells by regulating the expression of key proteins and their associated signalling pathways. For example, growth arrest-specific homeobox inhibits the proliferation of PASMCs by blocking the ERK 1/2 pathway, causing apoptosis of PASMCs via the Bcl-2/Bax pathway in hypoxia [150]. Furthermore, epigenetic factors (e.g., DNA methylation, interference by microRNAs and lncRNAs, and histone modification) also disturb the genetic landscape and expression of targeted mRNA/signalling, which causes aberrant biological processes and pathological alterations. In addition, dysregulation of PASMCs and HPAECs might be directly responsible for the pulmonary vascular remodelling observed in PAH, including abnormal proliferation, migration, apoptosis, autophagy and EndMT. Furthermore, increasing attention was placed on the role of metabolic dysfunction (oestrogen metabolism, mitochondrial dysfunction and glucose metabolism) in the development and progression of PAH. Particularly with respect to the controversial “oestrogen paradox”, greater efforts are needed to clarify whether oestrogen exerts positive or negative effects in PAH in patients and animal models. Increased glycolysis and mitochondrial fission also result in abnormal proliferation of vascular cells. Third, we presented approximately 25 types chemical ingredients isolated from natural medicines that have been found to protect against PAH in both in vitro and in vivo experiments, including alkaloids, flavonoids, polyphenols, glycosides, terpenes, quinones and polysaccharides. These natural medicines’ pharmacological effects are largely mediated through inhibition of PASMC proliferation, promotion of PASMC apoptosis, regulation of vasomotor factors, restraint of oxidative stress, attenuation of inflammatory response and attenuation of autophagy, which occur by modulating BMPR2/Smad, HIF-1α, PI3K/Akt/mTOR, eNOS/NO/cGMP, NF-κB and NLRP3 signalling pathways. Meanwhile, a broad spectrum of pharmacological experiments has indicated that microRNA also regulates proliferation, migration, apoptosis related signalling pathways through activating specific targets. Finally, we discuss the first-line treatment drugs of PAH, which are still phosphodiesterase type 5 inhibitors, endothelin receptor antagonists and prostacyclin analogues, such as sildenafil and bosentan. Moreover, most clinical trials focus on the abovementioned analogous therapeutic agents. However, extremely limited clinical evidence exists about natural medicines for the treatment of PAH. A small cohort study investigating TMP only implied a potential protective effect in PAH. Meanwhile, other clinical trials for PAH have exposed the vast heterogeneity among clinical protocols, for example, the divergent end-points, with a corresponding lack of specific biomarkers or targets. Despite recent progress, there are still gaps and doubts about PAH that need to be investigated by future studies.

First, although the five categories of PH all exhibit consistent pulmonary vascular remodelling caused by different factors, the features of PAH pathology show remarkable pulmonary vascular resistance induced by stenosis and occlusion of the pulmonary artery. Due to the non-specific clinical symptoms of PAH, i.e., shortness of breath, fatigue, weakness, angina and syncope, establishing appropriate methods to render a precise PAH clinical diagnosis would be of great significance to early therapeutic interventions. In addition to conventional pulmonary haemodynamics indexes (mPAP, PAWP, PVR), echocardiography has been recommended as the preferred clinical discriminator for suspected pulmonary hypertension patients. Meanwhile, other patient findings, such as history, physical examination, chest radiography and computed tomography assessment, should also be considered to comprehensively identify the correct PH category [151]. Thus, the specific pathological features of PAH should be marked to distinguish other PH subtypes to accurately define the clinical diagnosis of PAH. In addition, most research of physiological and pathological changes in pulmonary vessels concentrate on independent PASMCs or HPAECs, ignoring the adventitial fibroblasts. Moreover, investigation of the interaction between PASMCs and HPAECs in hypoxia/PDGF is also only rarely reported and is thought to be connected with pulmonary vascular remodelling. Only one study has demonstrated that co-culture of PASMCs and HPAECs unexpectedly promoted endothelial regeneration through BMPR2-Notch1-mediated glycolysis [64]. Hence, further investigation is needed on the interactions among PASMCs, HPAECs and fibroblasts in PAH, particularly with respect to physiopathology. Some scholars even posit that PAH might be a systemic disease involving neuroinflammation, the autonomic gut and its microbiota, and abnormal bone marrow cell trafficking. Subsequently, the hypothesis of “brain–gut–lung” interaction in PAH pathophysiology has been proposed [25].

Second, to date, cumulative results have indicated that genetic mutations, DNA methylation, interference of microRNAs and lncRNAs, and histone modifications all play crucial roles in the pathogenesis of PAH. But how to modulate target gene transcription and translation to protein remains unclear. Additionally, investigation regarding variations in mutated gene in the diagnosis, treatment and prognosis of PAH patients are still needed to examine these findings through the lens of advanced genomics. For example, utilizing the differences in genome-wide RNA expression profiling in lung tissue between PAH and other patients with distinct forms of PH will identify distinct gene expressions signatures involving biological function and mechanisms of pathophysiological [152]. Meanwhile, the “oestrogen paradox” remains a puzzle regarding the role of endogenous and exogenous oestrogen in PAH. This is due to the followed contradictory findings in clinical and experimental studies of PAH: (a) Results of epidemiological research suggest that PAH morbidity is higher in female than in male patients, yet the prognosis and survival rate of female PAH patients exhibits an obvious advantage. In other words, endogenous oestrogen might participate in the development of PAH by unknown mechanisms. Strangely, endogenous oestrogen gradually transforms into a beneficial effect in PAH after therapeutic interventions. Therefore, we deduce that endogenous oestrogen metabolism and signalling might be silently regulated by unknown methods during the progression of PAH, and sex differences might also be related to the prognosis and survival of PAH patients by influencing right ventricular function. Thus, consciously tracking dynamic variations of oestrogen in the diagnosis, therapy and prognosis of PAH patients in clinical trials is likely to help us understand the endogenous oestrogen confounding effects. For instance, a comparative study on the effects of proliferation in oestrogen-stimulated PASMCs derived from normal men and women revealed that oestrogen-driven attenuated BMPR-2 signalling in normal female PASMCs might be responsible for the prevalence of PAH in female patients [153]. Furthermore, the potential mechanism was likely due to ERα binding to the BMPR2 promoter to block its expression [154]. In addition, previous clinical research implied that the survival rate in female PAH patients compared to male might be attributed to the remarkably improved right ventricular ejection fraction (RVEF) in female patients [155]. The RVEF index is primarily used to evaluate survival in PAH patients. Hence, more maladaptive right ventricular remodelling is more prognostic in male PAH patients. In addition, another clinical study examining cardiovascular disease also reported that men exhibited reduced RVEF despite their greater right ventricular mass and volumes than women [156]. (b) Oestrogen also displays dramatically inconsistent outcomes in different PAH animal models. Female animals exhibit attenuated PAH compared to males in PAH models induced by hypoxia/MCT. Certain research has unexpectedly demonstrated the opposite results in specific PAH models (i.e., serotonin transporter overexpression, S100A4/Mts1^+^ transgenic mice and dexfenfluramine-induced PAH) [157–159]. Frump et al. investigated whether sex differences exist in PAH rats induced by Sugen5416/hypoxia and whether oestrogen exerts positive effects on RV function. Results indicated that female rats with ovariectomy experienced exacerbated right ventricular hypertrophy and cardiac index under Sugen5416/hypoxia compared to normal female rats. In contrast, the deteriorating RV function was improved by supplementary exogenous oestrogen [160]. Thus, exogenous oestrogen might possess protective effects in PAH. However, endogenous oestrogen exerts deleterious effects in serotonin transporter overexpression, S100A4/Mts1^+^ transgenic and dexfenfluramine-induced PAH female mice. Meanwhile, research results have demonstrated that endogenous oestrogen is involved in the pathogenesis of PAH in females in the same animal model [161]. Additionally, female mice with serotonin transporter overexpression also developed PAH via ERα-mediated oestrogen metabolic pathways. The elevated serotonin levels caused by the increased serotonin transporter enhanced CYP1B1 activity to promote proliferation of PASMCs [162]. In short, female gender may be a high-risk factor for PAH susceptibility because of disturbances in oestrogen metabolism. Thus, E2, androgens (dehydroepiandrosterone and testosterone), progestin and other sensitive compounds (e.g., serotonin, oestrogen metabolites and cytochrome P450) that participate in the oestrogen metabolism and dependent ER and BMP signalling should also be considered in the pathogenesis and treatment of PAH. Specifically, the potential interrelation should be investigated in the setting of the complex environment of PAH, i.e., hypoxia, gender, heritable variation, distribution of ER, variation of sex hormones, and intervention measure et al. [163, 164]. Moreover, owing to the limitation of MCT-induced mild endothelial injury in PAH animal not being consistent with the typical clinical characteristics, including prominent endothelial injury, stenosis and occlusion of pulmonary vessel and deposition of collagen. Therefore, multiple appropriate PAH animal models need to be established to better confirm the efficacy of PAH therapeutic agents through simulating the complex pathological characteristics that occur in the human disease. Further study should be conducted to disabuse the “contradiction” in endogenous and exogenous oestrogen observed by clinical studies and animal experiments. Furthermore, the aforementioned “oestrogen paradox” should consider novel evidence that has indicated that inflammation and infiltration of immune cells are also implicated in the development and progress of PAH, which might identify novel therapeutic approaches for PAH.

Third, limited research on natural medicines for the management of PAH have currently been reported, mostly covering alkaloids, flavonoids, polyphenols, glycosides, terpenes, quinones and polysaccharides. Pulmonary vascular remodelling and the haemodynamic index were significantly improved by these medicines in MCT/hypoxia-induced PAH animals. The potential mechanisms have been clarified by in vitro PASMC or HPAEC experiments. The results suggest that the above active ingredients exert protective effects in PAH by resisting PASMC proliferation, enhancing PASMC apoptosis, regulating the imbalance in vasomotor factors, and inhibiting oxidative stress and inflammation, which occur due to the regulation of BMPR2-Smad, HIF-1α, PI3K/Akt/mTOR, eNOS/NO/cGMP and MAPK/NF-κB pathways. Besides, limited and single PAH animal models may not yet provide sufficient evidence to support the efficacy of natural medicines, which may also contribute to the termination of clinical trials. Moreover, pharmacokinetic studies and safety evaluations of natural medicines in animals are only rarely reported. Therefore, subsequent studies should emphasize the gaps in enhancing validation of efficacy in multiple PAH animal models, performing necessary toxicity experiments. Additionally, investigation of natural medicines and their regulation of crucial genes and proteins in signalling pathway axes and binding sites to promoters at the genetic level still need to be clarified via dual-luciferase reporter and quantitative chromatin immunoprecipitation assay. Meanwhile, the important roles of microRNA and lncRNAs in the pathogenesis of PAH have been demonstrated by previous research [34, 165]. Hence, whether and how the active natural ingredients mediate microRNA and lncRNA function for regulating downstream signalling should also be further investigated. In addition to the aforementioned natural drug therapies, increasing novel and promising therapeutic approaches have been identified, such as enhancing apoptosis of PASMCs and HPAECs, targeting microRNA and lncRNAs, stem cell-based therapies and epigenetic medicines, or even gene transfer [31, 166]. In addition, despite abundant clinical research of prostacyclin analogues, endothelin receptor antagonists and phosphodiesterase type 5 inhibitors in the management of PAH are ongoing, but some clinical trial protocols are less rigorous, including unsuitable end-points without consideration of biomarkers according to the biological characteristics of PAH progression [167]. Furthermore, to date, there are limited reports about natural medicines for the treatment of PAH. Hence, the clinical study progress of natural drugs treating PAH should be driven by pharmacokinetics and pharmacodynamics with precise clinical design according to cumulative knowledge at the time.

**Fig. 7:**
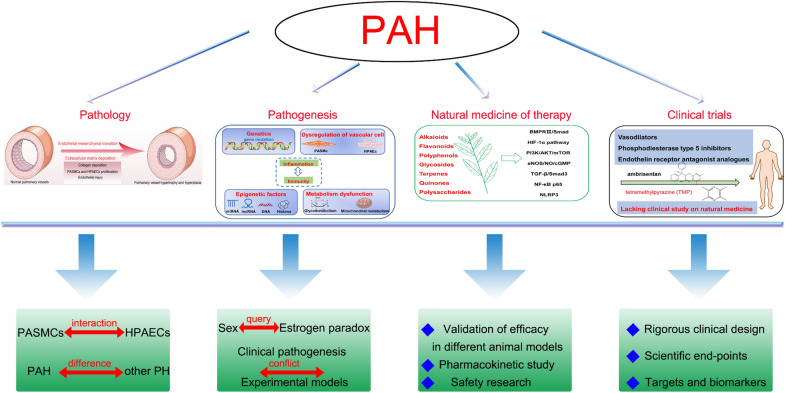
Comprehensive outline and new strategies on PAH. The interaction between PASMCs and HPAECs in hypoxia is also rarely reported and should be payed attention. Meanwhile, the specific pathological features of PAH should be marked to distinguish other PH subtypes to accurately define the clinical diagnosis of PAH. In pathogenesis, the “oestrogen paradox” should be further clarified regarding the role of endogenous and exogenous oestrogen in PAH. The “conflict” of oestrogen observed by clinical studies and animal experiments should also be investigated. Subsequent studies should emphasize the gaps in enhancing validation of efficacy in multiple PAH animal models, and pharmacokinetic studies and safety evaluations of natural medicines in animals are conducted in next study. Clinical study about natural medicines for the treatment of PAH should design scientific end-points, suitable targets and biomarkers

In conclusion, advancing knowledge on the pathology, pathogenesis, natural medicine therapies and mechanisms, and clinical study of PAH have been comprehensively summarized to identify potential new strategies. As described in Fig. 7, further study is needed to address existing issues and gaps with respect to PAH.

## Data Availability

Not applicable.

## Abbreviations

PAH: Pulmonary arterial hypertension
PH: Pulmonary hypertension
PAWP: Pulmonary artery wedge pressure
PVR: Pulmonary vascular resistance
NO: Nitric oxide
ET-1: Endothelin-1
mPAP: Mean pulmonary arterial pressure
HPAH: Hypoxic pulmonary artery hypertension
TSOC: Taiwan Society of Cardiology
CTEPH: Chronic thromboembolic pulmonary hypertension
BMPR2: Bone morphogenetic protein receptor 2
PASMCs: Pulmonary arterial smooth muscle cells
HPAECs: Human pulmonary arterial endothelial cells
RV: Right ventricular
EndMT: Endothelial–mesenchymal transition
ALT: Alanine aminotransferase
AST: Aspartate aminotransferase
TGF-β: Transforming growth factor- β
ACVRL1: Activin A receptor type II-like 1
ENG: Endoglin
KCNK3: Potassium channel subfamily K member 3
EIF2AK4: Eukaryotic translation initiation factor 2 alpha kinase 4
HIF: Hypoxia-inducible factor
BMP: Bone morphogenetic protein
ERK: Extracellular-signal-regulated kinase
MAPK: Mitogen-activated protein kinase
TRPC: Transient receptor potential cation channel
ROS: Reactive oxygen species
ER: Estrogen receptor
VEGF: Vascular endothelial growth factor
PECAM1: Platelet endothelial cell adhesion molecule 1
α-SMA: α-Smooth muscle actin
MCT: Monocrotaline
PDGF: Platelet-derived growth factor
E2: 17β-Estradiol
CYP1B1: Cytochrome P450 1B1
PFKFB3: 6-Phosphofructo-2-kinase/fructose-2,6-bisphosphatase 3
Notch1: Notch homolog 1
ILK: Integrin-linked kinase
N1ICD: Notch1 intracellular domain
Drp1: Dynamin-related protein 1
SOD: Superoxide dismutase
lncRNAs: Long noncoding RNAs
HDAC: Histone deacetylases
TNF: Tumor necrosis factor
IL: Interleukin
IgG: Immunoglobulin G
MMP: Matrix metalloproteinase
NF-κB: Nuclear factor-κB
PKG-1: Protein kinase type 1
NOS: Nitric oxide synthase
PPARγ: Peroxisome proliferator-activated receptor-γ
PI3K: Phosphatidylinositol 3-kinase
TrkA: Tyrosine receptor kinase A
PCNA: Proliferating cell nuclear antigen
FoxO_1_: Forkhead box protein O_1_
HMGB1: High mobility group box-1
STS: Sodium tanshinone IIA sulfonate
mTOR: Mammalian target of rapamycin
eIF2α: Eukaryotic initiation factor 2α
LV: Left ventricle
S: Septum
A_2a_R: Adenosine A_2a_ receptor
AMPKα1: Adenosine monophosphate-activated protein kinase α-1
Bax: Bcl-2-like protein 4
Bcl2: B-cell lymphoma 2
cGMP: Cyclic guanosine monophosphate
NLRP3: Recombinant NLR family: pyrin domain containing protein 3
SOCCs: Store-operated Ca^2^^+^ channels
eNOS: Endothelial nitric oxide synthase
ETA: Endothelin receptor A
NADPH: Nicotinamide adenine dinucleotide phosphate
SOCE: Store-operated Ca^2^^+^ entry
iNOS: Inducible nitric oxide synthase
MDA: Malondialdehyde
LC3B: Microtubule-associated protein 1A/1B light chain 3B
BECN-1: Beclin-1
ATG5: Autophagy protein 5
SQSTM1: Sequestosome-1
CDK4: Cyclin-dependent kinase 4
NT-proBNP: N-terminal pro brain natriuretic peptide
RVSP: Right ventricular systolic pressure
CAV1: Caveolin 1
RVHI: Right ventricular hypotrophy index
PDE5: Phosphodiesterase type 5
NALP3: NACHT, LRR and PYD domains-containing protein 3
miRNAs: MicroRNAs
FOXO3: Forkhead box O3
PERP: P53 apoptosis effector related to PMP‐22
TLR4: Toll-like receptor 4
PDCD4: Programmed cell death protein 4
SPRY2: Sprouty 2
PRKG1: Protein kinase cGMP-dependent type I
PTBP1: Polypyrimidine tract binding protein
PKM2: Pyruvate kinase M2
RVH: Right ventricular hypertrophy
SCs: Stem cells
RhoA-ROCK: Ras homolog family member A-Rho kinase
TMP: Tetramethylpyrazine
6MWD: 6-Minute walk distance
TTCW: Time to clinical worsening
RVEF: Right ventricular ejection fraction
